# Mutations in ALK signaling pathways conferring resistance to ALK inhibitor treatment lead to collateral vulnerabilities in neuroblastoma cells

**DOI:** 10.1186/s12943-022-01583-z

**Published:** 2022-06-10

**Authors:** Mareike Berlak, Elizabeth Tucker, Mathurin Dorel, Annika Winkler, Aleixandria McGearey, Elias Rodriguez-Fos, Barbara Martins da Costa, Karen Barker, Elicia Fyle, Elizabeth Calton, Selma Eising, Kim Ober, Deborah Hughes, Eleni Koutroumanidou, Paul Carter, Reda Stankunaite, Paula Proszek, Neha Jain, Carolina Rosswog, Heathcliff Dorado-Garcia, Jan Jasper Molenaar, Mike Hubank, Giuseppe Barone, John Anderson, Peter Lang, Hedwig Elisabeth Deubzer, Annette Künkele, Matthias Fischer, Angelika Eggert, Charlotte Kloft, Anton George Henssen, Michael Boettcher, Falk Hertwig, Nils Blüthgen, Louis Chesler, Johannes Hubertus Schulte

**Affiliations:** 1 Department of Pediatric Oncology/Hematology, Charité – Universitätsmedizin Berlin, Augustenburger Platz 1, 13353 Berlin, Germany; 2 Berlin School of Integrative Oncology (BSIO), Augustenburger Platz 1, 13353 Berlin, Germany; 3 Department of Clinical Pharmacy and Biochemistry, Institute of Pharmacy, Freie Universität Berlin, Kelchstr.31, 12169 Berlin, Germany; 4 Paediatric Solid Tumour Biology and Therapeutics Team, Clinical Division and Cancer Therapeutics Division, The Institute of Cancer Research, 15 Cotswold Road, Sutton, Surrey, SM2 5NG UK; 5 Otto Warburg Laboratory Gene Regulation and Systems Biology of Cancer, Max Planck Institute for Molecular Genetics, Berlin, Germany; 6 Institute of Pathology, Charité-Universitätsmedizin Berlin, 10117 Berlin, Germany; 7 IRI Life Sciences, Humboldt University Berlin, 10115 Berlin, Germany; 8 Experimental and Clinical Research Center (ECRC) of the Charité and Max-Delbrück-Center for Molecular Medicine (MDC) in the Helmholtz Association, 13125 Berlin, Germany; 9 Princess Maxima Center for Pediatric Oncology, Utrecht, The Netherlands; 10 Molecular Diagnostics Department, The Institute of Cancer Research and Clinical Genomics, The Royal Marsden NHS Foundation, London, UK; 11 Cancer Section, UCL Great Ormond Street Institute of Child Health, London, UK; 12 Department of Experimental Pediatric Oncology, Center for Molecular Medicine Cologne, 50931 Cologne, Germany; 13 Department of pharmaceutical sciences, Utrecht University, Utrecht, The Netherlands; 14 Department of Pediatric Hematology and Oncology, University Hospital, Tübingen, Germany; 15 German Cancer Consortium (DKTK), Berlin, Germany; 16 German Cancer Research Center (DKFZ), 69120 Heidelberg, Germany; 17 Berlin Institute of Health (BIH) at Charité—Universitätsmedizin Berlin, 10117 Berlin, Germany; 18 Medical Faculty, Martin Luther University Halle-Wittenberg, Halle (Saale), 06120 Halle, Germany

**Keywords:** Neuroblastoma, CRISPR screening, ALK, Resistance, NF1, NRAS, Trametinib, Lorlatinib, Ceritinib, Collateral sensitivity

## Abstract

**Background:**

Development of resistance to targeted therapies has tempered initial optimism that precision oncology would improve poor outcomes for cancer patients. Resistance mechanisms, however, can also confer new resistance-specific vulnerabilities, termed collateral sensitivities. Here we investigated anaplastic lymphoma kinase (ALK) inhibitor resistance in neuroblastoma, a childhood cancer frequently affected by activating ALK alterations.

**Methods:**

Genome-wide forward genetic CRISPR-Cas9 based screens were performed to identify genes associated with ALK inhibitor resistance in neuroblastoma cell lines. Furthermore, the neuroblastoma cell line NBLW-R was rendered resistant by continuous exposure to ALK inhibitors. Genes identified to be associated with ALK inhibitor resistance were further investigated by generating suitable cell line models. In addition, tumor and liquid biopsy samples of four patients with *ALK*-mutated neuroblastomas before ALK inhibitor treatment and during tumor progression under treatment were genomically profiled.

**Results:**

Both genome-wide CRISPR-Cas9-based screens and preclinical spontaneous ALKi resistance models identified NF1 loss and activating NRAS^Q61K^ mutations to confer resistance to chemically diverse ALKi. Moreover, human neuroblastomas recurrently developed de novo loss of *NF1* and activating RAS mutations after ALKi treatment, leading to therapy resistance. Pathway-specific perturbations confirmed that *NF1* loss and activating RAS mutations lead to RAS-MAPK signaling even in the presence of ALKi. Intriguingly, *NF1* loss rendered neuroblastoma cells hypersensitive to MEK inhibition.

**Conclusions:**

Our results provide a clinically relevant mechanistic model of ALKi resistance in neuroblastoma and highlight new clinically actionable collateral sensitivities in resistant cells.

**Supplementary Information:**

The online version contains supplementary material available at 10.1186/s12943-022-01583-z.

## Background

The development of molecular targeted therapies has significantly improved survival in a subset of cancer patients [1]. However, initial good responses to targeted therapies are frequently followed by resistance development [2, 3], preventing curative treatment in most cases using single-agent treatments. Once resistance develops, treatment options are often lacking. Intriguingly, some alterations conferring resistance to targeted therapies can also lead to new vulnerabilities, specifically in resistant cells, a concept termed collateral sensitivity [4].

Anaplastic lymphoma kinase (ALK) is a receptor tyrosine kinase frequently altered in cancers, either through chromosomal translocations leading to fusion of the ALK kinase domain with the amino-terminus of other proteins, or through activating point mutations or focal gene amplifications. *ALK* fusion genes drive tumorigenesis across a variety of different malignancies including anaplastic large cell lymphoma (ALCL) [5], non-small-cell lung cancer (NSCLC) [6] and inflammatory myofibroblastic tumor (IMT) [7]. Treatment with small molecule inhibitors for ALK, like ceritinib or lorlatinib, can be effective in a subset of patients, and ALK inhibitors have since entered routine and first-line therapy for many cancer entities [8, 9]. As observed with other targeted therapies, resistance to ALK inhibitors frequently occurs in *ALK*-driven cancer. While mechanisms leading to ALK inhibitor resistance in cancers with *ALK* fusion genes have been extensively studied [10], the mechanisms in tumors containing activating ALK mutations or *ALK* amplifications are largely unknown.

Neuroblastoma, a childhood tumor originating from the sympathetic nervous system [11], is a prototypical example of a cancer with recurrent *ALK* mutations. At diagnosis, up to 15% of neuroblastomas harbor activating point mutations or amplifications of *ALK*, and *ALK* mutations are further enriched upon disease relapse [12–20]. Mutated *ALK* is a driving oncogene in neuroblastoma, and neuroblastoma cells have a persistent and strong dependency on mutated ALK [21]. Activating mutations most often occur in the kinase domain, leading to increased ALK downstream signaling via the PI3K/AKT, RAS/MAPK and JAK/STAT pathways, promoting neuroblastoma cell survival and proliferation [14–19, 22–24]. Despite multimodal therapy including chemotherapy, surgery, radiation therapy and immunotherapy [25], high-risk neuroblastoma, often harboring *ALK* alterations [26], remains very difficult to treat [20, 27–29].

The initial optimism that ALK inhibitors could improve neuroblastoma outcome has been tempered by our recent understanding that the second most prevalent *ALK* mutation in sporadic neuroblastoma, ALK F1174L is intrinsically resistant to the first-generation ALK inhibitor, crizotinib [30, 31]. In contrast, phase I trials with the second-generation ALK inhibitor, ceritinib, showed clinically relevant responses in a fraction of patients with refractory or relapsed neuroblastoma, but the response duration was short, indicating early resistance development [32]. The third-generation ALK inhibitor, lorlatinib, is currently being evaluated to treat relapsed neuroblastoma in a phase I/II trial [33], and may soon be introduced into first-line therapy trials. Thus, understanding ALK inhibitor resistance mechanisms in neuroblastoma is of utmost clinical importance.

Only few mechanisms of ALK inhibitor resistance have been detected in preclinical models of neuroblastoma so far, most of which were adaptive epigenetic or gene expression changes that have not yet been observed in patients developing resistance and may not be therapeutically actionable [34–36]. Here, we aimed to identify clinically relevant genetic mechanisms of ALK resistance and collateral sensitivities resulting from such alterations. We recurrently detected *NF1* loss-of-function mutations as well as activating mutations in *RAS* and its analogues in clinical samples from ALK inhibitor-resistant neuroblastomas and confirmed the causal contribution of these mutations to ALK inhibitor resistance in preclinical models. Moreover, we identified hypersensitivity to MEK inhibition as a novel collateral sensitivity specific to *NF1* loss, which may represent a clinically actionable therapeutic strategy for ALK-inhibitor resistant neuroblastoma.

## Methods

### Cell lines

The human neuroblastoma cell lines Kelly (#ACC 355, female), SH-SY5Y (#ACC 209, female) and LAN-5 (#ACC 673, male) were obtained from DSMZ. NBLW-R were provided by the university of Chicago on behalf of Susan Cohn. All cell lines were cultured in a RPMI 1640-based medium (Life Technologies, #21875035). For NBLW-R this medium was supplemented with 10% fetal calf serum (FCS Superior, Sigma, #S0615) and for SH-SY5Y and Kelly with an additional 1% penicillin/streptomycin (Gibco, #15140122). For LAN-5 the medium was supplemented with 20% FCS, 1% penicillin/streptomycin, 1x non-essential amino acids (Roth, #9185.1) and 1x GlutaMAX (Gibco, #35050061). *NF1* knockout single cell clones were cultured under the same conditions as the respective parental cell line. All cell lines were incubated at 37 °C and 5% CO_2_. Lorlatinib-resistant NBLW-R.L2 and ceritinib-resistant NBLW-R.C1 were generated by exposure to increasing concentrations (20 nM to 10 μM*)* of lorlatinib or ceritinib for a time of 3 months. Both cell lines were cultured as the parental line and 20 nM lorlatinib or 20 nM ceritinib added respectively. SH-SY5Y TR were cultured in RPMI 1640, 10% FCS, 1% penicillin/streptomycin and 5 μg/ml Blasticidin (Invitrogen, #R210–01). For SH-SY5Y TR NRAS^Q61K^ cells 0.4 mg/ml G418 (Genaxxon, #M3118.0050) was added as well. Cell line identities of SH-SY5Y and LAN-5 cell lines and their respective *NF1* knockout single cell clones as well as for Kelly and SH-SY5Y TR were confirmed by STR profiling at the DSMZ. Cell lines were regularly tested for Mycoplasma using the PlasmoTest kit (InvivoGen, #rep-pt1). HEK293FT (female) were purchased from Thermo Fisher (#R7007) and cultivated in DMEM (Gibco, #61965026) according to manufacturer instructions. For details on cell line model generation please see Additional file 6 supplementary methods.

### Chemicals

Lorlatinib, ceritinib, trametinib, rapamycin and pictilisib were purchased from Axon Medchem (#Axon2600, #Axon224, #Axon1761, #Axon2069, #Axon1377). Lorlatinib was given from Pfizer for in vivo studies with the NBLW-R. LY3009120 was bought from Selleck Chemicals (#S7842).

### CRISPR-Cas9 knockout screen

For the CRISPR-Cas9-based negative selection screens performed in the neuroblastoma cell line SH-SY5Y, the human CRISPR knockout library Brunello was used as a one-vector system (Addgene, #73179) [37]. The pooled plasmid library targeting 19,114 genes with 76,441 sgRNAs (average of 4 sgRNAs per gene) was amplified as described elsewhere [37]. For details on library amplification and lentiviral production of the pooled plasmid library please also see Additional file 6 supplementary methods. To achieve the integration of one sgRNA per cell an MOI of 0.3 was used. To maintain a 1000x representation of each sgRNA at the timepoint of transduction 7 × 36.4*10^6^ SH-SY5Y cells were transduced per T300 flasks. 24 hours post transduction medium was changed. 48 hours post transduction puromycin selection medium (0.8 μg/ml puromycin, Thermo Fisher, #A1113803) was added to the cells and selection stopped after 6 days. Positively selected cells were expanded for 14 days to perform the screen with a ~ 1000x coverage. At d_0_ one sample was harvest as baseline sample (t_0_) and the other samples treated in two technical replicates with DMSO, ceritinib (0.3 μM, Axon Medchem, #2224) or lorlatinib (0.1 μM, Axon Medchem, #2600) for a total of 13 days. Medium was changed every third day. Cells were harvested on day 13 with a coverage of at least ~500x per condition. Genomic DNA (gDNA) was isolated using the ZymoResearch Quick-gDNA MidiPrep (ZymoResearch, #D4075). PCR amplification and high-throughput sequencing for sgRNA quantification are described below. Sequencing data was analyzed using MAGeCK-VISPR [38].

### Quantification of sgRNAs

To confirm the maintenance of the library representation after amplification of the pDNA pool, the library was amplified by PCR (cycling conditions: 1 × 1 min at 95 °C, 28 cycles of 30 s at 95 °C, 30 s at 53 °C, 30 s at 72 °C and 1 × 10 min 72 °C) using P5 primer mix and P7 A01 (Additional file 5 Table S5) as described elsewhere [39]. After gel extraction using the NucleoSpin Gel and PCR-Clean Up kit (Macherey-Nagel, #740609.50) the sample was also bead purified using the AMPure XP PCR purification protocol (Beckman coulter, #63880). After quality control experiments the sample was submitted for sequencing using the paired-end 150 MiniSeq Mid Output kit (Illumina, #FC-420-1004) with 10% PhiX. After confirmation of library representation, the pDNA pool was used for virus production. Genomic DNA of samples of the CRISPR/Cas9-based negative selection screen with ALK inhibitors was isolated as described above and used for PCR to quantify sgRNAs. A total of 26 μg genomic DNA per condition were used to perform 12 PCR reactions per sample to maintain a good representation (per reaction: 2.2 μg gDNA, 1 μM of primer mix P5, 1 μM of specific P7 primer per condition, 50 μl of Ultra II Q5 Master Mix Polymerase (NEB, #M0544L) and water for a total reaction volume of 100 μl). PCR cycling conditions: 1 × 1 min at 95 °C, 28 cycles of 30 s at 95 °C, 30 s at 53 °C, 30 s at 72 °C and 1 × 10 min 72 °C. PCR products were gel purified using the NucleoSpin Gel and PCR-Clean Up kit (Macherey-Nagel, #740609.50). Samples were sequenced as a pooled library using a NextSeq 550 sequencer, the paired-end 150 High Output kit (Illumina, #20024907) and 10% PhiX. The sequences of the primers used for PCR analyses are described in Additional file 5 Table S5.

### RT-qPCR

RNA was isolated with TRIzol™ Reagent (Invitrogen, #15596026) according to manufacturer guidelines. To determine mRNA expression 1 μg or 500 ng RNA were incubated for 10 min at 65 °C with oligo (dT)_18_ primer and transcribed into cDNA using the transcriptor first strand cDNA synthesis kit (Roche, #04379012001) (55 °C for 30 min, 50 °C for 1 hour, 85 °C for 5 min). For qPCR performance on a QuantStudio™3 Real-Time PCR System (Applied Biosystems™, #A28567) a 1:4 dilution of cDNA was mixed with 5 μl FastStart Essential DNA Green Master (Roche, #06402712001) and primers (Additional file 5 Table S5). Quantitative PCR cycling conditions: 50 °C for 2 min,95 °C for 10 min, [95 °C for 15 s, 60 °C for 1 min, 95° for 15 s] (40 cycles), 60 °C for 1 min and 95 °C for 15 s. For each sample technical duplicates were prepared and a total of three biological replicates.

### Drug treatments and cell viability measurement

For inhibitor treatments the cell lines Kelly, LAN-5 (5000 cells/well), SH-SY5Y (3000 cells/well), NBLW-R, NBLW-R.LR, NBLW-R.CR (each 10,000 cells/well) as well as SH-SY5Y TR and respective model systems were seeded in white 96-well plates 24 hours before the inhibitor treatment. Inhibitors dissolved in DMSO were applied using a Tecan D300e digital dispenser and each concentration was added with 3 technical replicates. Cell viability assessed after 72 hours (or 10 days for NBLW-R, and 5 days for NBLW-R.LR or NBLW-R.CR) using the ATP quantification assay CellTiter-Glo® (Promega, #G7571) according to manufacturer protocol. To determine cell numbers during and after 72 hours of inhibitor treatment experiments with the live-cell imaging system Incucyte® S3 (Sartorius) were performed. Therefore, LAN-5, SH-SY5Y as well as SH-SY5Y TR and respective model systems were seeded according to their specific growth rate in clear 96-well plates 24 hours before the inhibitor treatment (LAN-5 15,000 cells/well, LAN-5 *NF1* KO#1 17,000 cells/well, LAN-5 *NF1* KO#2 17,000 cells/well, SH-SY5Y 15,000 cells/well, SH-SY5Y *NF1* KO#1 19,000 cells/well, SH-SY5Y *NF1* KO#2 8000 cells/well, SH-SY5Y TR EV and respective NRAS^Q61K^ clones 8000cells/well). For each well 4 images using a 10x objective were taken and analyzed using the cell-by-cell module. For each experiment at least 3 biological replicates were conducted. Evaluation of data and generation of concentration-response curves was performed using GraphPad Prism 9.0 and the four-parameter logistics model.

### Perturbation experiments and computational modelling

Respective cell lines were seeded, and serum starved for 24 hours. Cells were exposed to different inhibitors, ceritinib (SH-SY5Y: 600 nM, LAN-5: 337 nM), lorlatinib (LAN-5: 478 nM), trametinib (SH-SY5Y: 49 nM, LAN-5: 31 nM), pictilisib (SH-SY5Y: 1 μM, LAN-5: 31 nM), rapamycin (SH-SY5Y and LAN-5: 100 nM) or DMSO for 90 minutes. Subsequently cells were stimulated with 25 ng/ml EGF (R&D systems, #AFL236–200), 100 ng/ml IGF-1 (R&D systems, #291-G1–200) or PBS for 30 minutes. Cells were harvested using cell scrapers on ice and lysed using the Bio-Plex Pro Cell signaling reagent kit (Bio-Rad, #171304006 M). Subsequently lysates were incubated with antibody-coated magnetic beads as described elsewhere [40]. Beads were specific for P-AKT (S473), P-ERK1/2 (Thr202/Tyr204/Thr185/Tyr187), P-MEK1 (S217/S221) and P-S6K (Thr389). Samples were analyzed using the Bio-Plex MAGPIX multiplex reader (Bio-Rad). Result files were further analyzed using the R package LXB (https://cran.r-project.org/web/packages/lxb/index.html) and a custom script to generate MIDAS-formatted files (following *DataRail* [41]). Subsequently perturbation data was used for computational modeling using the R package STASNet [42] (https://github.com/molsysbio/STASNet).

### Western blot for treatment

Samples for western blot analysis of ALK downstream signaling during inhibitor treatment were prepared the following: cells were seeded, and serum starved for 24 hours. SH-SY5Y cells were treated with 49 nM trametinib (LAN-5: 31 nM), 600 nM ceritinib (LAN-5: 337 nM) or DMSO for a total 1 hour which included 30 minutes of stimulation with 25 ng/ml EGF (R&D systems, #AFL236–200) EGF or PBS (Carrier). NBLW-R cells were seeded, and serum starved for 24 hours, then treated with 100 nM of either ceritinib, lorlatinib, trametinib or DMSO for 1 hour which included 30 minutes of stimulation with 25 ng/ml EGF or PBS. Cells were harvested on ice using a cell scraper in cell lysis buffer (15 mM HEPES, 150 mM NaCl, 10 mM EDTA, 2% Triton X-100, pH 7.5,1xPhosSTOP,1xcOmplete Mini EDTA free protease inhibitor, 10 μg/ml Leupeptin, 10 μg/ml Aprotinin, 200 μM PMSF, 25 mM NaF) [43]. Determination of protein concentration, SDS-PAGE as well as semi-dry blots were performed as described in Additional file 6 supplementary methods.

### Illustration tool

Graphical schemes were created with BioRender.

### Quantification and statistical analysis

GraphPad Prism 9.0 was used to generate concentration response curves and to perform statistical analyses. Statistical tests used are specified in the Figure legends. Error bars represent mean ± SD unless otherwise indicated in the figure legends.

For details on plasmid library amplification, lentivirus production and transduction, cell line model generation, protein lysate preparation and western blot, in vivo studies, whole-exome sequencing of cell lines, high-throughput drug screen, droplet digital PCR of NBLW-R cells, panel sequencing of tumor samples and panel sequencing of cfDNA samples, please see Additional file 6 supplementary methods.

## Results

### A genome-wide CRISPR knockout screen identifies *NF1* as a mediator of ALK inhibitor sensitivity

**Fig. 1:**
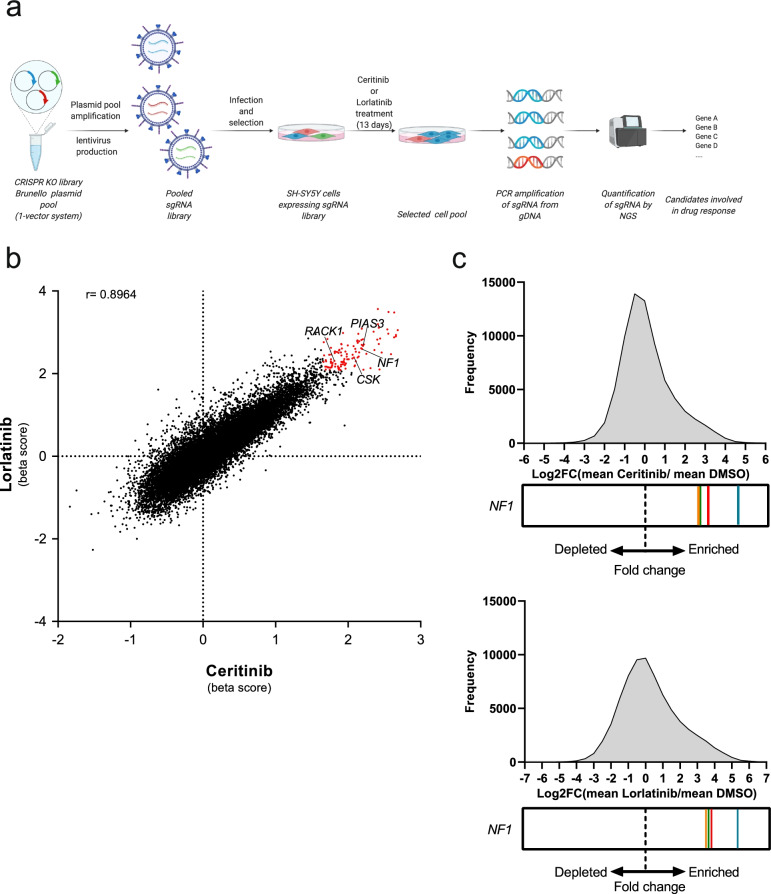
Genome-wide CRISPR/Cas9 knockout screens identify genes associated with ALK inhibitor response. **a** Schematic of CRISPR/Cas9 knockout screens in the neuroblastoma cell line SH-SY5Y. **b** Overview of screen results. Negative beta scores indicate depletion of sgRNAs targeting denoted genes during treatment, whereas positive beta scores indicate enrichment. Significant candidate genes (*P* < 0.01) with highly abundant sgRNAs during treatment are highlighted in red; Pearson correlation coefficient *r* = 0.896, *p* < 0.0001 (two-tailed t-test). **c** Histograms depicting the abundance distribution of all sgRNAs to their log_2_ fold change (mean ALK inhibitor/mean DMSO) for both ALK inhibitors. The enrichment of sgRNAs targeting *NF1* is shown below, with each colored line representing one of the four sgRNAs targeting *NF1*. The enrichment of sgRNAs is consistent between both ALK inhibitors

To identify genes mediating an ALK inhibitor-resistant phenotype in neuroblastoma cells, we performed a genome-wide CRISPR-Cas9-based knockout screen in neuroblastoma cells incubated in the presence or absence of ALK inhibitors. Prior to screening, we investigated ALK inhibitor responses of ALK-mutated neuroblastoma cell lines harboring *MYCN* amplifications (Kelly^ALKF1174L^, LAN-5^ALKR1275Q^) or non-amplified *MYCN* (SH-SY5Y^ALKF1174L^) (Additional file 1 Fig. S1) to select a cell line and establish suitable ALKi concentrations for screening. The screen was performed in SH-SY5Y cells transduced with the genome-wide lentiviral single guide RNA (sgRNA) Brunello library [39] and with lorlatinib or ceritinib concentrations leading to 70 -80% reduction of growth for 13 days (Fig. 1a). These samples were compared to SH-SY5Y cells incubated with DMSO as a control. Abundance of sgRNA sequences were quantified by next-generation sequencing before (t_0_) and 13 days after treatment and read count matrices were used for MAGeCK-VISPR analysis [38]. Based on MAGeCK-VISPR ‘β’ scores, we identified 109 genes with significantly enriched gene-targeting sgRNAs (*P* ≤ 0.01) in cells treated with either ALK inhibitor (Fig. 1b). Technical screen replicates for each treatment showed a high correlation (r ≥ 0.98) of normalized counts per sgRNA (Additional file 1 Fig. S2a and S2b). According to the published literature, some of these genes were members of ALK downstream signaling pathways, such as the JAK/STAT pathway and Src signaling (Additional file 3 Table S3). Most interestingly, all sgRNAs targeting *NF1*, a Ras-GTPase activating protein (Ras-GAP) and negative regulator of Ras signaling [44], were among the most significantly enriched in cells treated with either ALK inhibitor (Fig. 1b and c), suggesting NF1 to be crucial for ALK inhibitor response. Thus, loss of proteins modulating ALK downstream signaling, and NF1 particularly, may lead to ALK inhibitor resistance.

### *NF1* knockout results in ALK inhibitor resistance

**Fig. 2:**
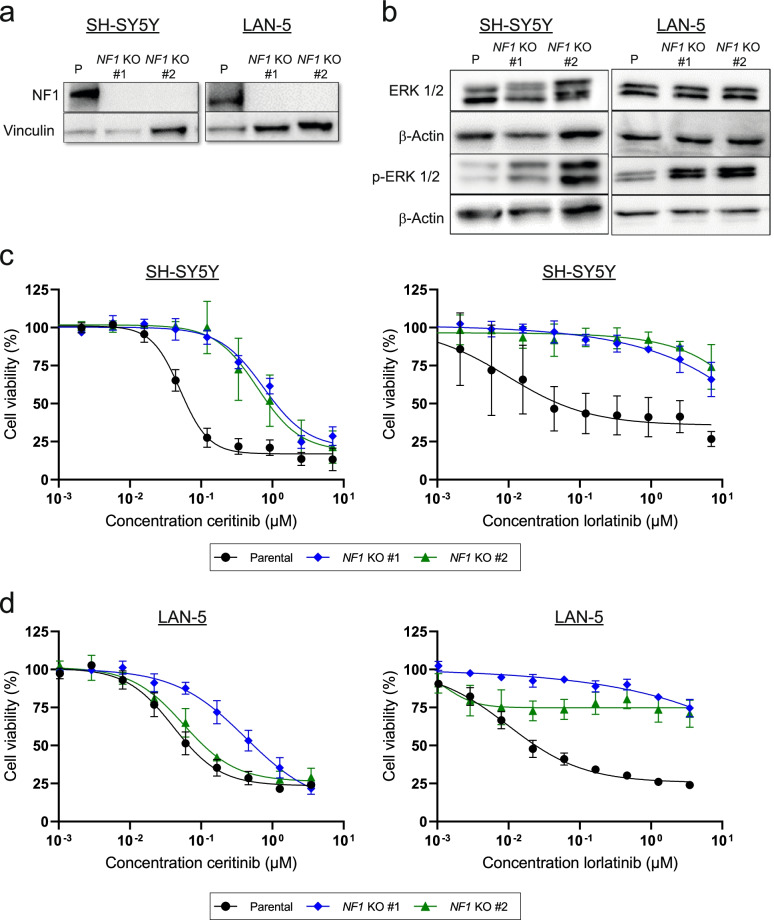
*NF1* knockout leads to ALK inhibitor resistance in neuroblastoma cell lines. **a** Knockout of *NF1* in different *ALK*-mutated neuroblastoma cell lines using CRISPR-Cas9 leads to an absence of NF1 protein. **b** Western blot analysis of total and phosphorylated ERK 1/2 indicates increased RAS/MAPK signaling in *NF1* knockout single-cell clones. **c** and **d** Cell viability of *NF1* knockout clones was assessed during ALK inhibitor treatment with ceritinib or lorlatinib and indicated decreased cell sensitivity; values represent mean ± SD, *n* = 3

To formally demonstrate that loss of *NF1* can cause ALK inhibitor resistance in neuroblastoma cell lines, we generated several neuroblastoma cell line models harboring *NF1* knockouts. We used the neuroblastoma cell lines SH-SY5Y (*ALK*^*F1174L*^) and LAN-5 (*ALK*^*R1275Q*^*, MYCN*-amplified), to represent ALK-mutated neuroblastoma within a genomic background either with or without *MYCN* amplification. The absence of mutations in ALK downstream signaling pathways was verified in these cell lines by targeted and exome sequencing. *NF1* knockouts were introduced using CRISPR-Cas9 targeting exon 1 or 30. After generating and validating isogenic cell lines for *NF1* knockout (Fig. 2a), we observed increased RAS/MAPK signaling as seen by an increased phosphorylation of ERK1/2 in *NF1* knockout clones (Fig. 2b), in line with NF1 function in this pathway [45]. Sensitivity towards ALK inhibition was significantly reduced in cells lacking NF1, as assessed by treatment of the cells with lorlatinib or ceritinib for 72 hours and determination of cell viability (Fig. 2c, d, Additional file 1 Fig. S7a and S7b). These data demonstrate that loss of *NF1* is sufficient to cause ALK inhibitor resistance in ALK-mutated neuroblastoma cell lines.

### *NRAS* mutations spontaneously arise during resistance development to ALK inhibitors

**Fig. 3:**
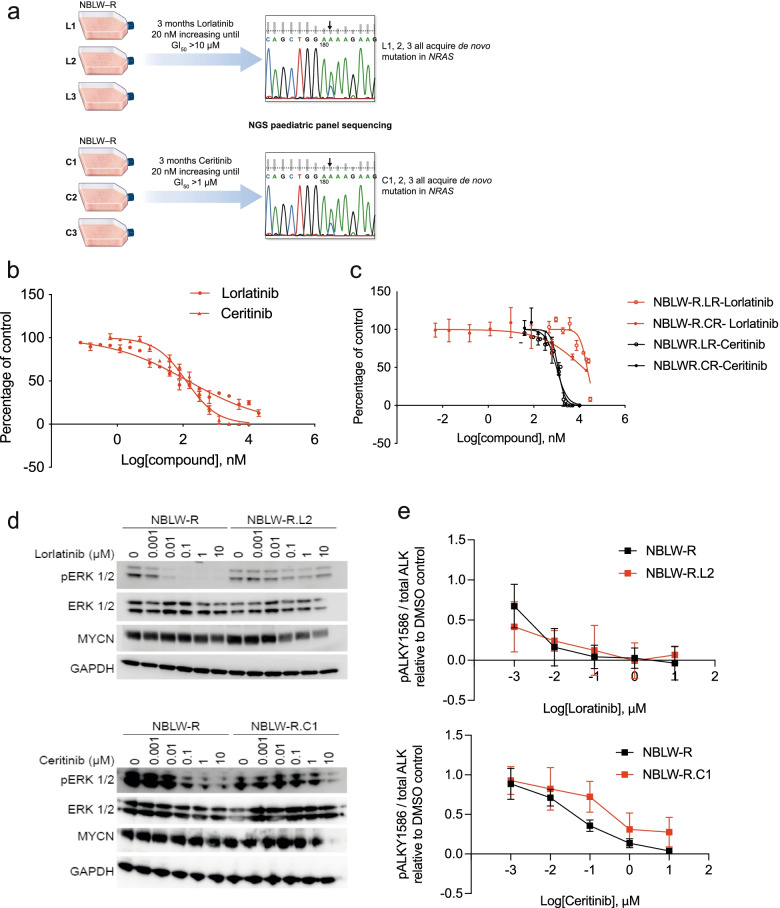
Continuous treatment with ALKis leads to de novo NRAS mutations and ALKi resistance in NBLW-R **a** Schematic to illustrate the induction of resistance to either lorlatinib or ceritinib in NBLW-R within 3 months. **b** and **c** 10-day GI_50_ of lorlatinib (0.073 μM) and ceritinib (0.109 μM) in NBLW-R parental line and 5-day GI_50_ of lorlatinib and ceritinib in lorlatinib-resistant NBLW-R (NBLW-R.LR (mean of L1, 2 and 3)) and ceritinib-resistant NBLW-R (NBLW-R.CR (mean of C1, 2 and 3)). NBLW-R.LR lorlatinib > 20 μM; NBLW-R.LR ceritinib > 900 nM; NBLW-R.CR lorlatinib 14 μM; NBLW-R.CR ceritinib > 1 μM. **d** and **e** Immunoblots and ALK immunoassay of cell lysates from NBLW-R versus NBLW-R.L2, and NBLW-R versus NBLW-R.C1 following treatment of cells with indicated ALKi for 1 hour, values represent mean ± SD, *n* = 3

In order to explore de novo mutations occurring during development of ALK inhibitor resistance, we cultivated NBLW-R neuroblastoma cells (*ALK*^*F1174L*^, *MYCN*-amplified) with increasing concentrations of lorlatinib or ceritinib for a period of 3 months (Fig. 3a) to generate resistant cell lines. Targeted sequencing was performed at the end of treatment, detecting recurrent *NRAS* mutations, c.C181 > A or c.A182 > G, both known to result in constitutively active RAS (NRAS^Q61K^ or NRAS^Q61R^ respectively) and increased signaling downstream of MAPK [46]. In addition, this was validated using droplet digital PCR (ddPCR) (Additional file 1 Fig. S3a). Each resistant cell line also demonstrated cross-resistance to alternative ALK inhibitors in comparison to the parental line, suggesting structure-independent resistance (Fig. 3b, c and Additional file 1 Fig. S3b). Activation of NRAS in these models was confirmed by western blotting for phosphorylated ERK1/2 following treatment with ALK inhibitors in resistant cells compared to the parental cell lines as controls. ERK1/2 remained phosphorylated in the resistant cell models even after treatment with higher concentrations of ALK inhibitors (Fig. 3d). Maintenance of ALK dephosphorylation after treatment was confirmed by immunoassay (Fig. 3e). MRI scans and growth analysis in an orthotopic kidney capsule murine model revealed that both lorlatinib- and ceritinib-resistant cells formed tumors more quickly than parental NBLW-R cells (Additional file 1 Fig. S3c and S3d). In line with our in vitro results, lorlatinib or ceritinib treatment of mice engrafted with ceritinib-resistant NBLW-R lines demonstrated significantly reduced efficacy of lorlatinib compared with mice engrafted with parental NBLW-R cells (Additional file 1 Figure S3e). These data suggest that both NF1 loss and acquisition of activating NRAS mutations, either of which can activate MAPK signaling, can induce ALK inhibitor resistance.

### Expression of mutant NRAS leads to ALK inhibitor resistance

**Fig. 4:**
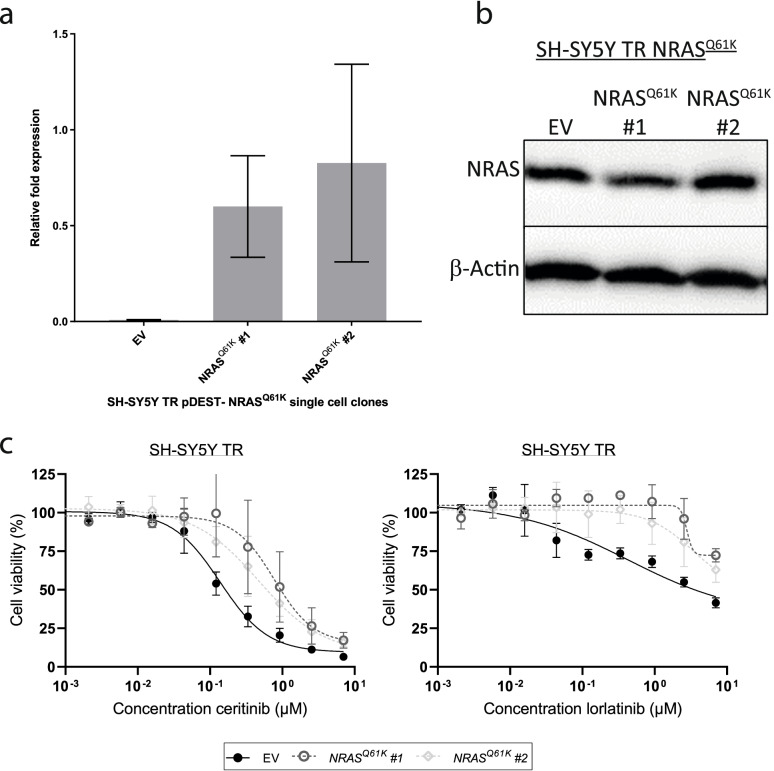
Mutated NRAS^Q61K^ causes ALK inhibitor resistance in ectopic expression model. **a** Ectopic expression of mutated *NRAS* (NRAS^Q61K^) measured using a mutation specific qPCR without tetracycline treatment; values represent mean ± SD, *n* = 3. **b** Western blot analysis of total NRAS indicates slightly increased NRAS protein levels in SH-SY5Y TR NRAS^Q61K^ clones without induction with tetracycline. Note that the antibody is not mutation-specific, but detects both, ectopically expressed mutant NRAS as well as endogenously expressed wildtype NRAS. **c** Cell viabilities of SH-SY5Y TR NRAS^Q61K^ clones and empty vector control were assessed during ALK inhibitor treatment with ceritinib or lorlatinib indicating an ALKi resistant phenotype; values represent mean ± SD, *n* = 3

To investigate whether the NRAS^Q61K^ mutant, which arose in cells spontaneously developing ALK inhibitor resistance, is sufficient to induce ALK inhibitor resistance, we generated SH-SY5Y cells inducibly expressing NRAS^Q61K^ (tetracycline-dependent). We analyzed *NRAS* expression in two independent clonal cultures as well as an empty vector control cell clone, using western blotting and RT-qPCR. RT-qPCR revealed strong induction of NRAS^Q61K^ by tetracycline treatment (Additional file 1 Fig. S4). In line with the results of RT-qPCR, western blot experiments revealed strong induction of NRAS by tetracycline treatment, with western blot being unable to discriminate between wildtype NRAS and mutated NRAS^Q61K^ (data not shown). However, significant expression of NRAS^Q61K^ was detected by mutation-specific RT-qPCR also in the absence of tetracycline compared to the empty vector control (Fig. 4a). This suggested a significant leakiness of our expression system, rendering comparisons of clones in the absence and presence of tetracycline less effective. Instead, we compared cells expressing NRAS^Q61K^ to the empty vector control cells in the absence of tetracycline. While NRAS^Q61K^ was clearly expressed in the NRAS^Q61K^–transfected clones in the absence of tetracycline (as detected by mutation specific RT-qPCR) and was completely absent in the empty vector control cells, western blot analysis revealed that overall NRAS protein levels did not relevantly exceed baseline levels in the NRAS^Q61K^–transfected clones as compared to empty vector control cells (Fig. 4b), which allowed us to specifically analyze the effect of the presence of the Q61K mutation rather than the effect of excessive NRAS expression. Cells expressing NRAS^Q61K^ were significantly more resistant to either lorlatinib or ceritinib compared to isogenic control cells harboring the empty vector (Fig. 4c). This demonstrates that NRAS^Q61K^ mutation is sufficient to induce an ALK inhibitor resistant phenotype in neuroblastoma cell lines.

### *NF1* loss-of-function mutations and activating *RAS* mutations occur in ALK inhibitor-resistant relapsed human neuroblastomas

**Fig. 5:**
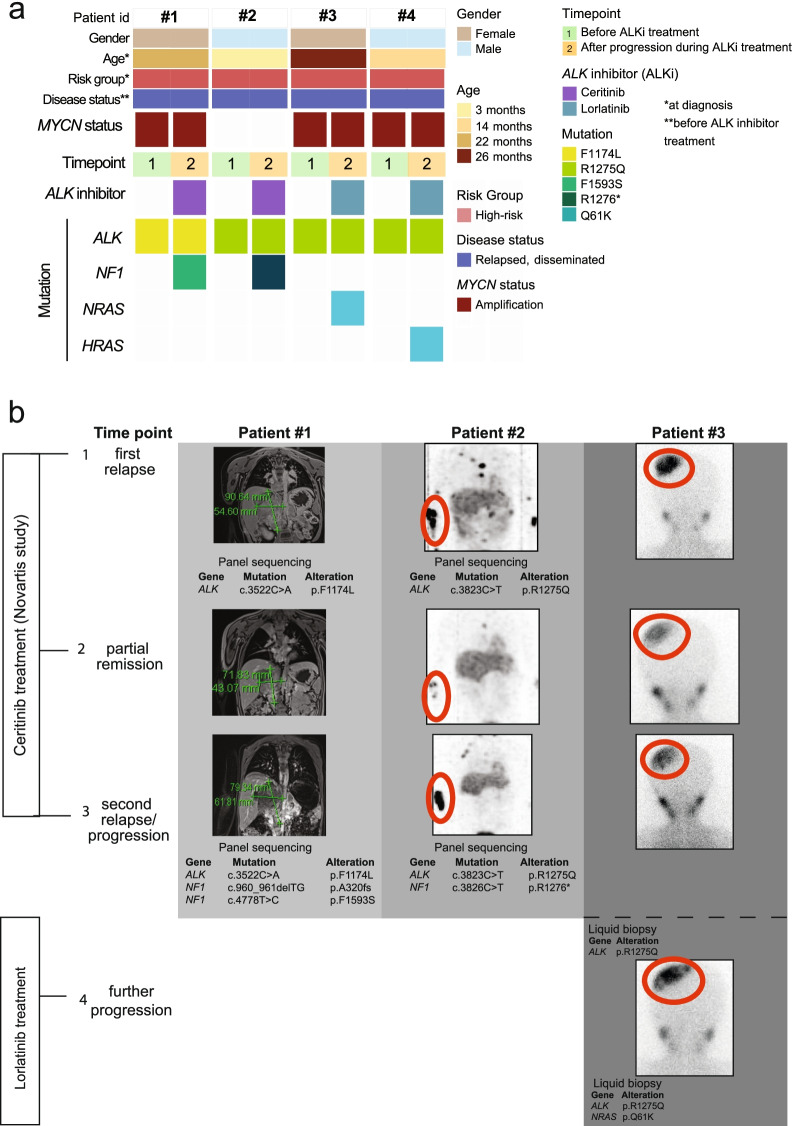
Mutations in ALK downstream signaling cause ALK inhibitor resistance in *ALK*-mutated neuroblastomaa Clinical covariates of the high-risk neuroblastoma cohort (*n* = 4) before and after development of ALK inhibitor resistance. **b** Magnetic resonance imaging (MRI) scans and Iodine-123 metaiodobenzylguanidine scintigraphy scans (MIBG) of patients whose tumors harbored ALK^R1275Q^ or ALK^F1174L^ mutations during treatment with ceritinib. After partial remission both, patient 1 and 2, relapsed under ceritinib treatment and de novo *NF1* mutations were detected using targeted sequencing. Tumor lesions are highlighted by red circles. **c** MIBG- scans of patient whose tumor harbored ALK^R1275Q^ mutations during treatment with ceritinib and lorlatinib. After partial remission the patient relapsed under ALKi treatment and de novo *NRAS* mutations were detected using targeted sequencing. Tumor lesions are highlighted by red circles

In order to investigate the clinical relevance of our observations that both NF1 loss and NRAS activation were sufficient to induce ALK inhibitor resistance in neuroblastoma models, we genomically profiled tumor and liquid biopsy samples (whole-exome sequencing or a hybrid-capture targeted next-generation sequencing assay) from four patients with neuroblastomas harboring activating *ALK* mutations before ALK inhibitor treatment and during tumor progression under treatment (Fig. 5a, also see Table S1). In line with our observations in preclinical models, known loss-of-function *NF1* mutations (NF1 ^R1276*^, NF1^A320fs^ and NF1^F1593S^) occurred de novo in two patients after treatment with ceritinib (Fig. 5b). In samples from two patients treated with lorlatinib we detected de novo *NRAS* (Fig. 5c) or *HRAS* mutations (NRAS^Q61K^ and HRAS^Q61K^), associated with constitutively activated RAS protein of the respective isoform [47, 48]. These observations further affirm the clinical relevance of *NF1* and *RAS* in ALK inhibitor resistance in neuroblastoma.

### Loss of NF1 causes increased RAS-MAPK signaling and diminished negative ERK-RAF feedback

**Fig. 6:**
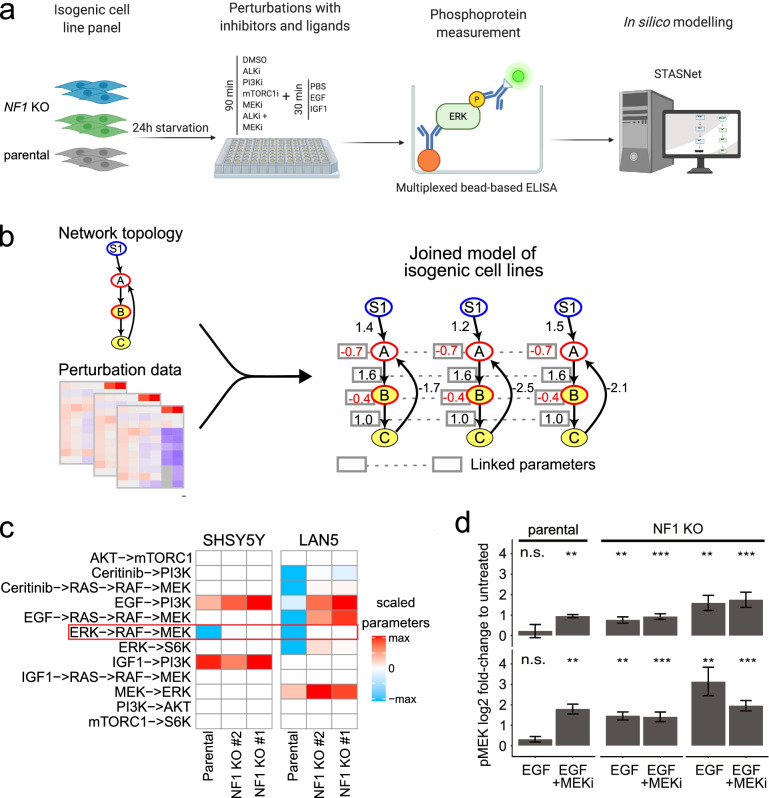
*NF1* knockout cell line models show increased RAS-MAPK signaling and a weakened ERK-RAF feedback. **a** Schematic of perturbation experiments performed in *NF1* knockout cell lines. **b** Schematic of computational modeling using STASNet. **c** Computational modeling of ALK downstream signaling using STASNet. Model paths strength shown as a heatmap based on relative values of scaled parameters with separate scaling for LAN-5 and SH-SY5Y cell lines when those parameter values are found to vary between cell lines*.* Negative feedback is indicated in blue. *NF1* knockout models show a weaker ERK-RAF inhibitory feedback in comparison to the respective parental cell line (red box). **d** Shown is the log-fold change of phosphorylated MEK level for the parental cell line and *NF1* knockout models stimulated with EGF or stimulated with EGF and exposed to MEK inhibitor, compared to an unperturbed control, values represent mean ± standard error of the mean, *n* = 3

To understand how loss of NF1 alters ALK signaling pathways and to potentially identify collateral sensitivities pointing toward new treatment options for patients with disease resistant to ALK inhibitors, we performed perturbation experiments and subsequent computational modeling of signaling networks using steady-state analysis of signaling networks (STASNet) [42]. *NF1* knockout cell models and their parental controls were exposed to inhibitors targeting ALK, MEK, PI3K or mTOR and subsequently stimulated with the growth factors, EGF or IGF1. Relative phosphorylation of signaling components downstream of ALK was measured using a multiplexed bead-based ELISA assay (Fig. 6a). As expected, NF1 loss was associated with increased RAS-MAPK signaling (see also Fig. 2b), in line with the role of NF1 as a negative regulator of RAS/MAPK signaling [45]. The perturbation-response data (Fig. S5) together with a prior knowledge network of the signaling topology served as input for the STASNet signaling network modeling pipeline (Fig. 6b). The output of the modelling procedure are quantified signaling interactions and differences due to the *NF1* knockout. More precisely, STASNet adjusts model parameters representing the strength of signaling interactions in the signaling network and inhibitor efficacy such that the model simulations fitted the data optimally. We constrained the model parameters of isogenic cell line triplets such that they were identical between the isogenic cell lines triplets and allowed divergence of parameters between these cell lines only if it was necessary to fit the data, as quantified by a likelihood ratio test. This procedure reflected that molecular changes between isogenic cell lines were minimal and that we expected that most molecule interactions remain quantitatively similar. When we inspected the model parameters that diverged, we noticed a weakened negative feedback from ERK to RAF in all *NF1* knockout clones in comparison to the parental cell lines (Fig. 6c, red box and Additional file 1 Fig. S5). Such a negative feedback restricts MAPK signaling in parental cells, and consequently EGF activated MEK only when MEK inhibitors were present in parental cell lines, whereas EGF activated MAPK signaling efficiently in *NF1* knockout cells irrespective of MEK blockage (Fig. 6d). Strong negative feedback, as we detected in the parental neuroblastoma cell lines, is a known resistance mechanism against MEK inhibitors since it results in an accumulation of phosphorylated MEK leading to reactivation of downstream targets [49, 50]. Taken together, these results suggest that MAPK signaling upon *NF1* knockout in neuroblastoma cells harboring ALK mutations is associated with loss of negative ERK-RAF feedback, leading us to hypothesize that these cell lines may be particularly sensitive to MEK inhibitor treatment.

### Deletion of NF1 in *ALK*-mutated neuroblastoma cells increases MEK inhibitor sensitivity

**Fig. 7:**
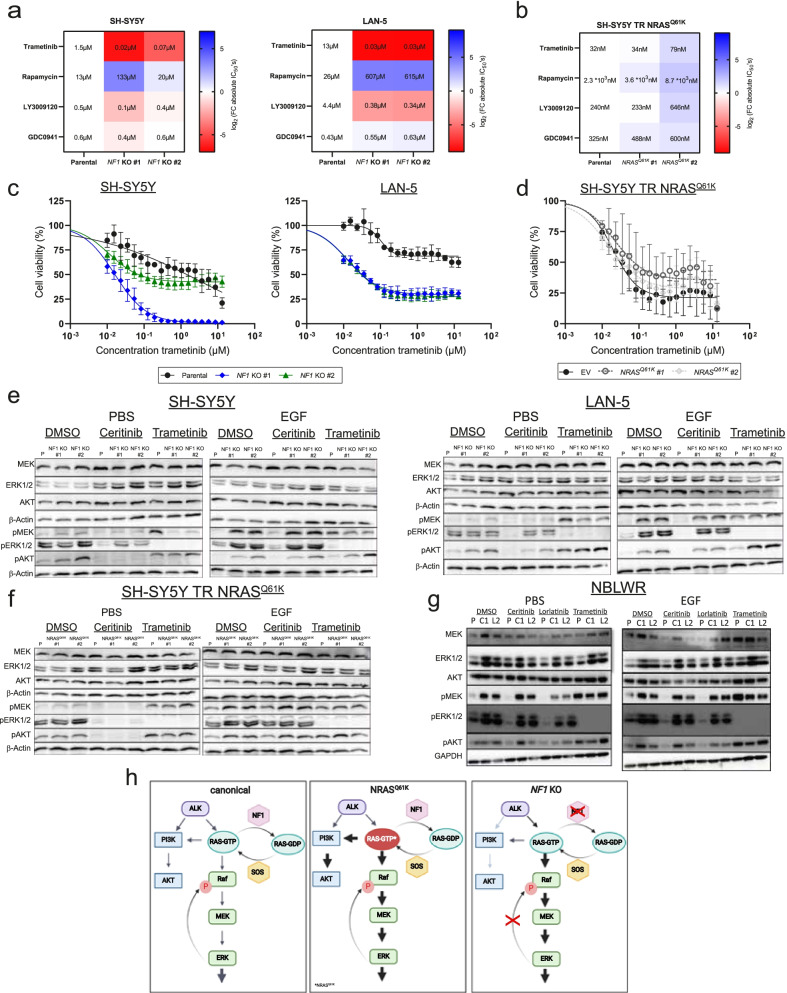
*NF1* knockout cell line models are sensitive to MEK and pan-RAF inhibitor treatment. **a** and **b** Drug screen of *NF1* knockout cell lines and ectopic NRAS^Q61K^ expression models. Cell were treated with trametinib (MEKi), rapamycin (mTORi), pictilisib (GDC0941, PI3Ki) or pan-RAF inhibitor LY3009120 for 72 hours and cell viability assessed using CellTiterGlo measurements. Colors indicate log2(FC) of absolute IC_50_ values to values of the parental or empty vector control cell lines. Red indicates a higher sensitivity in comparison to the parental cell line or empty vector control and blue a decreased sensitivity. Respective absolute IC_50_ values are shown for each treatment. **c** and **d** Respective concentration-response curves of *NF1* knockout clones and ectopic NRAS^Q61K^ expression models for MEK inhibitor treatment with trametinib; values represent mean ± SD, *n* = 3. **e**, **f** and **g** Western blot analysis of 24-hour-serum-starved *NF1* knockout cell lines, ectopic NRAS^Q61K^ expression models and NBLW-R resistant models exposed to DMSO, ceritinib, lorlatinib or trametinib for 1 hour with subsequent stimulation for 30 minutes with EGF or PBS. **h** Schematic of canonical ALK downstream signaling in comparison to signaling in neuroblastoma cell lines with mutated NRAS^Q61K^ or a *NF1* knockout

To test predictions derived from the computational modeling, we investigated MEK inhibitor sensitivity in *NF1* knockout models and ectopic NRAS^Q61K^ expression models by performing a small inhibitor screen for 4 inhibitors of signaling downstream of ALK. Indeed, *NF1* knockout clones were more sensitive than the parental cell line to MEK inhibition by trametinib as well as the pan-RAF inhibitor, LY3009120, (Fig. 7a and c). In contrast, sensitivity of *NF1* knockout clones towards the mTOR inhibitor, rapamycin, and the PI3K inhibitor, GDC0941, was unaltered or only slightly reduced from that in parental cell lines. *NF1* knockout cells, contrary to cells with ectopic NRAS^Q61K^, were not differentially sensitive (compared with control cell lines harboring the empty vector) to any of the drugs screened (Fig. 7b, d and Additional file 1 S6a). A high-throughput screen of 197 drugs was also performed to compare drug sensitivities between the LAN-5 *NF1* KO #1 knockout clone and its parental line, which revealed a singular and specific hypersensitivity of the *NF1* knockout clone to different MEK inhibitors that was not present in the parental line (Additional file 1 Fig. S7a and S7c). Responses to ALK and MEK inhibitors were investigated in western blots from all cell lines, detecting low levels of MEK phosphorylation (Ser217/221) and an absence of ERK phosphorylation in *NF1* knockout clones during trametinib treatment (Fig. 7e). These results were consistent with our computational modeling data, which indicated a weak or missing ERK-RAF feedback (Fig. 6d). Ectopic NRAS^Q61K^ expression (in comparison to *NF1* knockout clones) was associated with elevated MEK phosphorylation after EGF stimulation and ceritinib exposure. MEK phosphorylation in ectopic NRAS^Q61K^ expressing clones was comparable to levels detected for the empty vector control during trametinib treatment (Fig. 7f), indicating NRAS^Q61K^ expression did not alter ERK-RAF feedback. Responses of NBLW-R parental and resistant lines to trametinib further confirmed that de novo NRAS^Q61K^ acquisition seems to have no effect on the ERK to RAF feedback, as evidenced by their insensitivity to MEK inhibition and strong phosphorylated MEK levels after MEK inhibitor treatment (Fig. 7g). In conclusion, only *NF1* loss but not the expression of oncogenic NRAS^Q61K^ seems to trigger loss of feedback-mediated MEK reactivation and leads to increased sensitivity of neuroblastoma cell lines harboring *ALK* mutations to MEK inhibition. Our results suggest MEK inhibitor sensitivity is a new vulnerability and collateral sensitivity in a subset of ALK inhibitor-resistant neuroblastomas (Fig. 7h).

## Discussion

Clinical responses to targeted inhibitors often do not translate into improved patient cure rates due to frequent development of therapy resistance [51, 52]. Improving cure rates, therefore, depends on our understanding of resistance mechanisms and the development of treatment strategies for therapy-resistant cancers. Here, we analyzed ALK inhibitor resistance in neuroblastoma, and demonstrated that loss of NF1 or mutation of NRAS^Q61K^ can lead to ALK inhibitor resistance. We identified MEK inhibitor sensitivity as a collateral sensitivity of ALK inhibitor resistance due to NF1 loss in *ALK*-mutated neuroblastoma cells.

To determine mechanisms of ALK inhibitor resistance, we first screened preclinical models and subsequently analyzed tumor samples or liquid biopsies obtained from patients before and during ALK inhibitor treatment (before/after initial response and during disease progression). Preclinically, we performed two unbiased screens. A CRISPR/Cas9 knockout screen using two different ALK inhibitors, ceritinib and lorlatinib, determined genes associated with ALK inhibitor resistance. We identified genes encoding proteins regulating signaling downstream of ALK that were modulated by both ALK inhibitors, with the most interesting hit being *NF1*. In parallel, we generated resistant cell populations in an *ALK*-mutated neuroblastoma cell line through constant lorlatinib or ceritinib exposure. De novo NRAS^Q61K^ mutations arose but NF1 loss was not observed in resistant populations created in the continuous exposure resistance model. This in vitro response is in line with our observation that NF1 was lost and activating RAS mutations were acquired in samples from patients. The underlying determinants for these differences remain to be investigated, but our findings clearly suggest that the route to MAPK activation can differ.

Some recent publications also mainly reported mechanisms involving epigenetic rewiring or overexpression of receptor tyrosine kinases other than ALK, as mechanisms rendering neuroblastoma cells independent of ALK signaling [34–36], but their clinical relevance remained elusive to date. These mechanisms should be non-mutually exclusive with our observations, as they are driven by deregulated gene expression (e.g., *BORIS, AXL, PIM1*) rather than resistance mutations. It will be important to investigate different contributions of individual genetic and epigenetic mechanisms in future studies. It may be reasonable to speculate that some genetic mechanisms observed in our study may be a cause of deregulated gene expression observed in other studies. In line with our results, an independent report suggested NF1 alterations were potentially involved in ALK resistance [53] and a case report described a de novo NRAS^Q61K^ mutation upon development of ALK inhibitor resistance in a lorlatinib-treated neuroblastoma [54]. To our knowledge, our report is the first to extend these observations and provide mechanistic evidence for their causal relationship.

*NF1* knockout and ectopic NRAS^Q61K^ expression confirmed that these alterations can confer ALK inhibitor resistance, independent of cell line context or ALK mutation type. Resistance was more pronounced against lorlatinib than ceritinib. We believe that the ALK specificity and affinity of these two inhibitors may cause these differences, with lorlatinib being more specific for mutant ALK, while ceritinib is known to be less specific with more off-target cytotoxic effects [55]. Such off-target toxicity of ceritinib may reduce the effect of NF1 loss and NRAS^Q61K^ acquisition, compared to lorlatinib.

In line with our in vitro data, we detected de novo *NF1* and *NRAS* or *HRAS* mutations in neuroblastomas from patients treated with the ALK inhibitors, ceritinib or lorlatinib, at resistance development. Due to limited material from the respective tumors, the presence of relevant fractions of nonmalignant cells and technical limitations of the hybrid-capture panel sequencing analysis, it could not be unequivocally determined if a *NF1* wildtype allele was maintained or lost in the tumors of patients #1 and #2 after ALK inhibitor treatment. In addition, it remained elusive if the two *NF1* mutations detected in the tumor of patient #2 after ALK inhibitor treatment occurred in two separate clones or in one clone, and in the latter case if these mutations occurred in *cis* or in *trans*. It will be important to extend these findings to prospective studies, which will enable measurements of mutation incidence and a more detailed analysis of *NF1* alterations. Despite the small sample size, our observations in four independent patients provide strong evidence for the clinical significance of our preclinical findings, and represents, to our knowledge, the first analysis of this sort. Intriguingly, all neuroblastomas in patients treated with ceritinib acquired inactivating *NF1* alterations, whereas neuroblastomas in patients treated with lorlatinib acquired activating RAS mutations. We believe this unlikely to be due to mechanistic differences, but future prospective studies investigating such differences may be warranted.

While we report *NF1* and *RAS* mutations in the context of ALK resistance development in neuroblastoma, loss-of-function *NF1* mutations and activating *RAS* mutations, as well as other RAS-MAPK pathway-activating mutations have been observed in primary and relapsed neuroblastoma independent of ALK inhibitor treatment [20, 56, 57]. In contrast to our observations of *ALK* mutations co-occurring with NF1 or activating RAS mutations, these mutations were mutually exclusive in neuroblastoma samples from patients not treated with ALK inhibitors. This suggests that whereas MAPK pathway activation in primary tumors can occur through mutually exclusive ways, additional activating alterations can rescue the repressed RAS-MAPK activity in the presence of ALK inhibitors. It is reasonable to speculate that this re-activation might also occur through other mutations that affect MAPK activity, which should be tested in future trials.

Our findings are in line with reports of RAS-MAPK signaling reactivation through the loss of NF1 as a mechanism of resistance to other targeted therapies, including EGFR inhibition in lung cancer [58], BRAF inhibition in melanoma [59], BCR-ABL inhibition in chronic myeloid leukemia [60] and endocrine therapies in advanced breast cancer [61]. In contrast, activating NRAS^Q61K^ mutations have mainly been reported as arising in the context of BRAF inhibitor resistance in melanoma [62–64].

A yet often underappreciated strategy builds on the hypothesis that resistance mutations induce new vulnerabilities, depicted collateral sensitivities, and uses these new vulnerabilities to design serial combination therapies, thereby taking advantage of the process of resistance development rather than trying to counteract it. We perturbed and computationally modeled signaling networks in our *NF1* knockout models to identify new vulnerabilities and potential new treatment options. We were able to show that NF1 loss shifts signaling downstream from ALK from a broader distribution among the JAK/STAT, AKT/PI3K and RAS/MAPK pathways towards stronger signaling exclusively along the RAS-MAPK axis. Most importantly, modeling indicated that ERK-RAF feedback was weakened, which led us to predict MEK inhibitor sensitivity as a new collateral sensitivity that was confirmed in our small inhibitor screen in the *NF1* knockout cell line model. Most treatment-naïve neuroblastoma cells are only intermediately sensitive to MEK inhibitors [65], in line with the failure of MEK inhibitors in clinical trials to treat neuroblastoma [66]. Our *NF1* knockout models were, contrastingly, hypersensitive to MEK inhibitor treatment. Phosphorylated MEK and ERK 1/2 levels decreased during trametinib treatment in *NF1* knockout clones, whereas phosphorylated MEK levels increased in the respective parental lines, likely maintaining sufficient ERK activity for cell proliferation. The latter was also observed for our ectopic NRAS^Q61K^ expression model and ALK inhibitor-resistant NBLW-R cell populations. In this context, phosphorylated MEK levels during MEK inhibitor treatment correlate with the strength of the ERK to RAF feedback. Cell lines less responsive or resistant to MEK inhibitors have previously been shown to possess strong inhibitory ERK to RAF feedback that is abrogated upon MEK inhibition to result in even higher levels of phosphorylated MEK and reactivation of the pathway [49, 50]. Cell lines sensitive to MEK inhibitors, however, show only a weakened or absent negative ERK to RAF feedback, which we propose as the mechanistic reason for MEK inhibitor sensitivity in neuroblastoma cell lines lacking NF1. Accordingly, we recently demonstrated in work available on the bioRxiv preprint server that the strength of ERK to RAF feedback correlates with the level of MEK inhibitor sensitivity in neuroblastoma cell lines [65]. Surprisingly, even though NF1 and NRAS^Q61K^ mutations both increase RAS/MAPK signaling, thereby conferring ALK inhibitor resistance, cells harboring these alterations respond differently to MEK inhibitor treatment. This appears to be due to the different strengths of ERK to RAF feedback. The mechanism by which NF1 loss abrogates ERK to RAF feedback in neuroblastoma cells harboring ALK mutations remains elusive, but this will not prevent a rapid translation of this collateral sensitivity into the clinic. However, further investigation of the mechanism in the future may help to develop new ways of overcoming the feedback reactivating of the RAS/MAPK pathway in *NF1* wild type neuroblastomas to render them sensitive to MEK inhibitors in general.

## Conclusion

Our study identifies *NF1* loss and activating RAS mutations as bona fide clinically relevant causes of ALK inhibitor resistance in ALK-driven neuroblastoma and establishes reactivation of signaling downstream from the RAS-MAPK pathway as a mechanism of ALK inhibitor resistance. Extending the concept of collateral sensitivities, we identified MEK inhibitor hypersensitivity as a new vulnerability after NF1 loss in *ALK*-mutated neuroblastoma cells. This presents a potential treatment option for patients with neuroblastomas that have lost NF1 and are resistant to ALK inhibitors, with the potential to impact clinical practice and future clinical trial design.

## Supplementary Information

**Additional file 1: Figure S1:** ALK inhibitor treatment of ALK-mutated neuroblastoma cell lines for optimized screeningconditions (related to Figure 1). **Figure S2:** Quality control of CRISPR/Cas9 knockout screen (related to Figure 1). **Figure S3:** Lorlatinib- and Ceritinib- resistant NBLW-R neuroblastoma cells grow as aggressive tumors in the kidney capsule of nude mice (related to Figure 3). **Figure S4:** Tetracycline induced NRASQ61K expression (related to Figure 4). **Figure S5:** Computational modeling of ALK downstream signaling using STASNet (related to Figure 6). **Figure S6:** MEK inhibitor treatment of ectopic NRASQ61K expression models (related to Figure 7). **Figure S7:** High-throughput drug screening of LAN-5 and LAN-5 NF1 KO#2 clone (related to Figure 7). **Table S1.** Full clinical data on neuroblastoma patients.

**Additional file 2: Table S2.** Normalized read counts. CRISPR-Cas9 knockout screen-Normalized read counts (related to Figure 1).

**Additional file 3: Table S3.** MAGeCK VISPR analysis. CRISPR-Cas9 knockout screen-MAGeCK VISPR analysis (related to Figure 1).

**Additional file 4: Table S4.** Gene beta scores cutoff at p0.01. CRISPR-Cas9 knockout screen- Gene beta scores cutoff at p0.01 (related to Figure 1).

**Additional file 5: Table S5.** Oligonucleotides used. Oligonucleotides used in this study (related to Figures 1, 2 and 4).

**Additional file 6.** Supplementary Materials and Methods.

## Data Availability

The CRISPR-Cas9 knockout screen datasets analysed during the current study are available as FASTQ Files in the NCBI Sequence Read Archive (SRA) with the BioProject accession number PRJNA765129 and will be publicly available as of the date of publication (reviewer link: https://dataview.ncbi.nlm.nih.gov/object/PRJNA765129?reviewer=s6aulos3t1j2qhp94f44u19fe8). The whole exome sequencing (WES) dataset for the LAN-5 cell line generated and analysed during the current study is available as FASTQ Files in the Sequence Read Archive (SRA) repository with the BioProject accession number PRJNA765501 (https://www.ncbi.nlm.nih.gov/sra/?term=SRR16018268). The panel sequencing dataset for the cell line SH-SY5Y generated and analysed during the current study is available as FASTQ Files in Sequence Read Archive (SRA) with the BioProject accession number PRJNA772100 (https://www.ncbi.nlm.nih.gov/sra/?term=SRR16387283). The patient sequencing datasets of patients treated in Berlin are available in the European Genome-Phenome Archive (EGA) repository with the study number: EGAS00001005791 and dataset number: EGAD00001008343 (https://ega-archive.org/datasets/EGAD00001008343). The patient sequencing datasets of the patient treated in London, UK are available in the European Genome-Phenome Archive (EGA) repository with the study numbers: EGAS00001006163 and EGAS00001006162. The patient sequencing datasets of the patient treated in Cologne, Germany used during the current study are available from the corresponding author on reasonable request. Cell lines generated in this study have not been deposited but are available upon request, with restrictions to the availability of the neuroblastoma cell line NBLW-R as the IP is held by The University of Chicago. Further information, incucyte images and requests for resources and reagents should be directed to and will be fulfilled by the corresponding author, Johannes Hubertus Schulte (johannes.schulte@charite.de or johannes.schulte@uni-due-de).

## Abbreviations

ALCL: Anaplastic large cell lymphoma
ALK: Anaplastic lymphoma kinase
ALKi: Anaplastic lymphoma kinase inhibitor
cfDNA: Circulating free DNA
CRISPR: Clustered regularly interspaced short palindromic repeats
ctDNA: Circulating tumor DNA
ddPCR: Droplet digital PCR
EGF: Epidermal growth factor
ELISA: *Enzyme linked immunosorbent assay*
ERK1/2: Extracellular signal-regulated kinase 1/*2*
IGF1: Insulin-like growth factor 1
IMT: Inflammatory myofibroblastic tumor
MAGeCK: Model-based Analysis of Genome-wide CRISPR/Cas9 Knockout
MAPK: Mitogen-activated protein kinase
MEK: MAP 2K, Mitogen-activated protein kinase kinase
MRI: Magnetic resonance imaging
NF1: Neurofibromin 1
NSCLC: Non-small-cell lung cancer
pDNA: Plasmid DNA
RT-qPCR: Real-time *quantitative PCR*
sgRNA: Single guide RNA
STASNet: Steady-state analysis of signaling networks
