# Supplementary material for: Mutations in ALK signaling pathways conferring resistance to ALK inhibitor treatment lead to collateral vulnerabilities in neuroblastoma cells
Source: Mol Cancer. 2022 Jun 10;21:126. doi: 10.1186/s12943-022-01583-z (PMC9185889; doi:10.1186/s12943-022-01583-z)
Supplement: Supplementary file 1 — Additional file 1: Figure S1: ALK inhibitor treatment of ALK-mutated neuroblastoma cell lines for optimized screeningconditions (related to Figure 1). Figure S2: Quality control of CRISPR/Cas9 knockout screen (related to Figure 1). Figure S3: Lorlatinib- and Ceritinib- resistant NBLW-R neuroblastoma cells grow as aggressive tumors in the kidney capsule of nude mice (related to Figure 3). Figure S4: Tetracycline induced NRASQ61K expression (related to Figure 4). Figure S5: Computational modeling of ALK downstream signaling using STASNet (related to Figure 6). Figure S6: MEK inhibitor treatment of ectopic NRASQ61K expression models (related to Figure 7). Figure S7: High-throughput drug screening of LAN-5 and LAN-5 NF1 KO#2 clone (related to Figure 7). Table S1. Full clinical data on neuroblastoma patients. [file 12943_2022_1583_MOESM1_ESM.pdf]

Figure S1

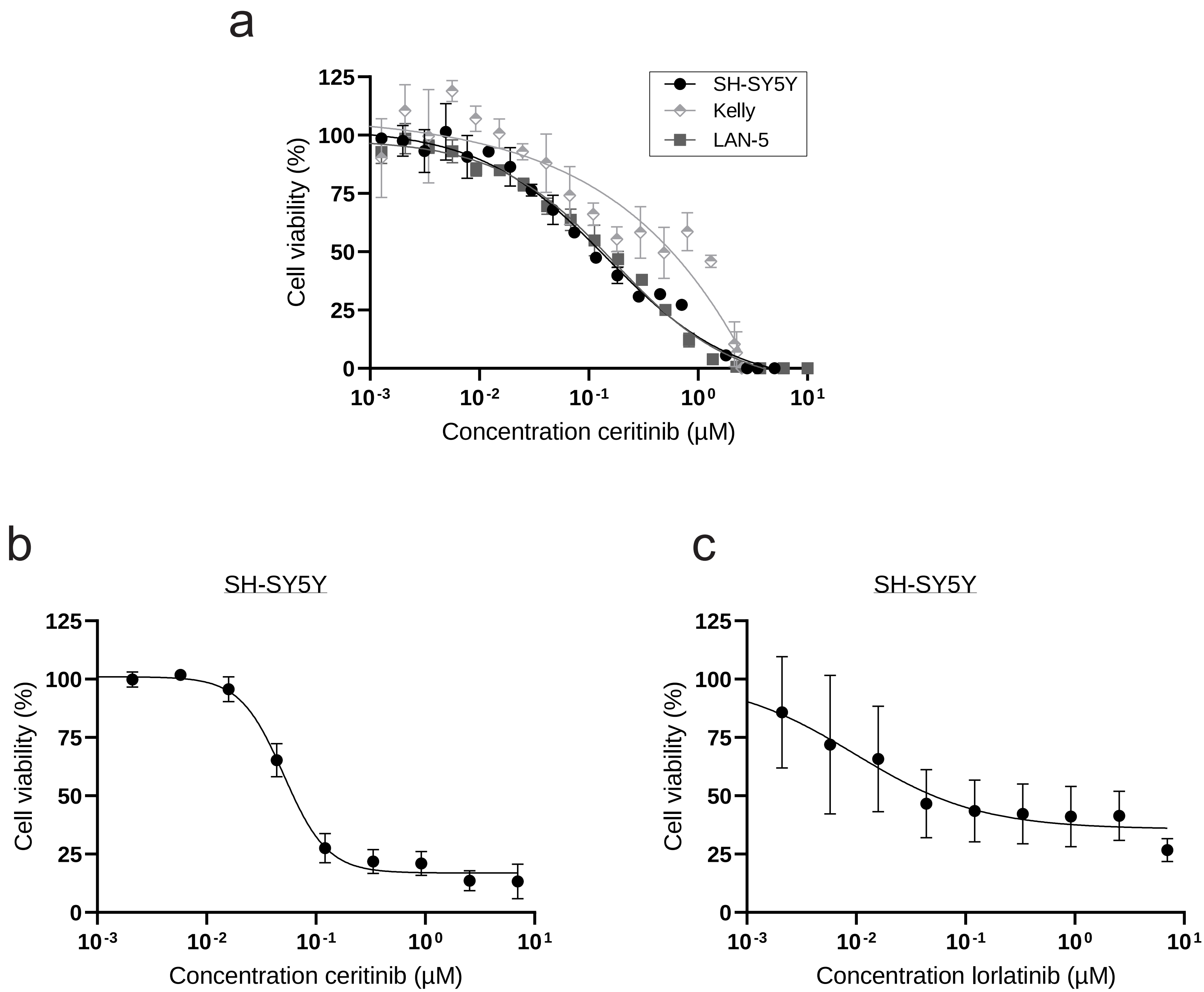

a
SH-SY5Y
Kelly
LAN-5
Cell viability (%)
Concentration ceritinib (µM)
b
SH-SY5Y
Cell viability (%)
Concentration ceritinib (µM)
c
SH-SY5Y
Cell viability (%)
Concentration lorlatinib (µM)

Figure S2

a

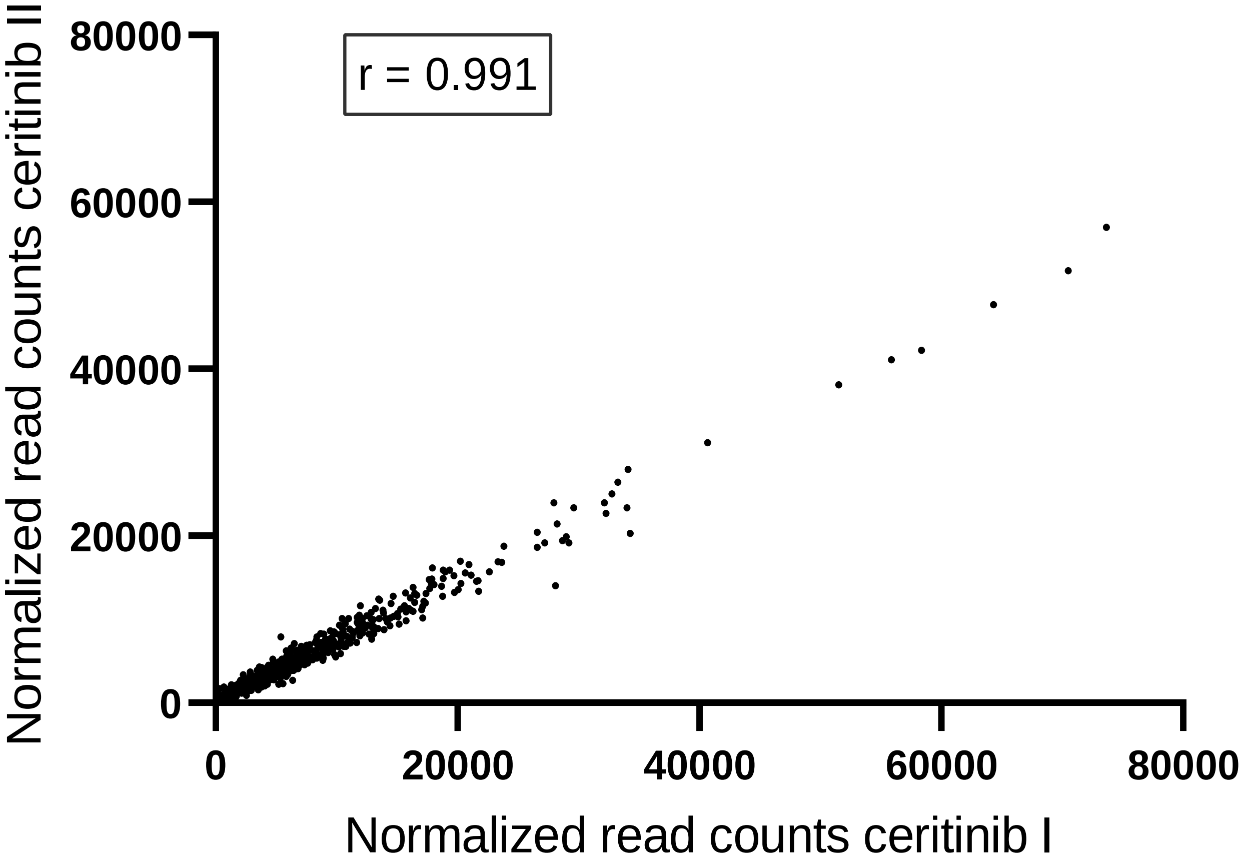
r = 0.991
Normalized read counts ceritinib II
Normalized read counts ceritinib I
0
20000
40000
60000
80000

b

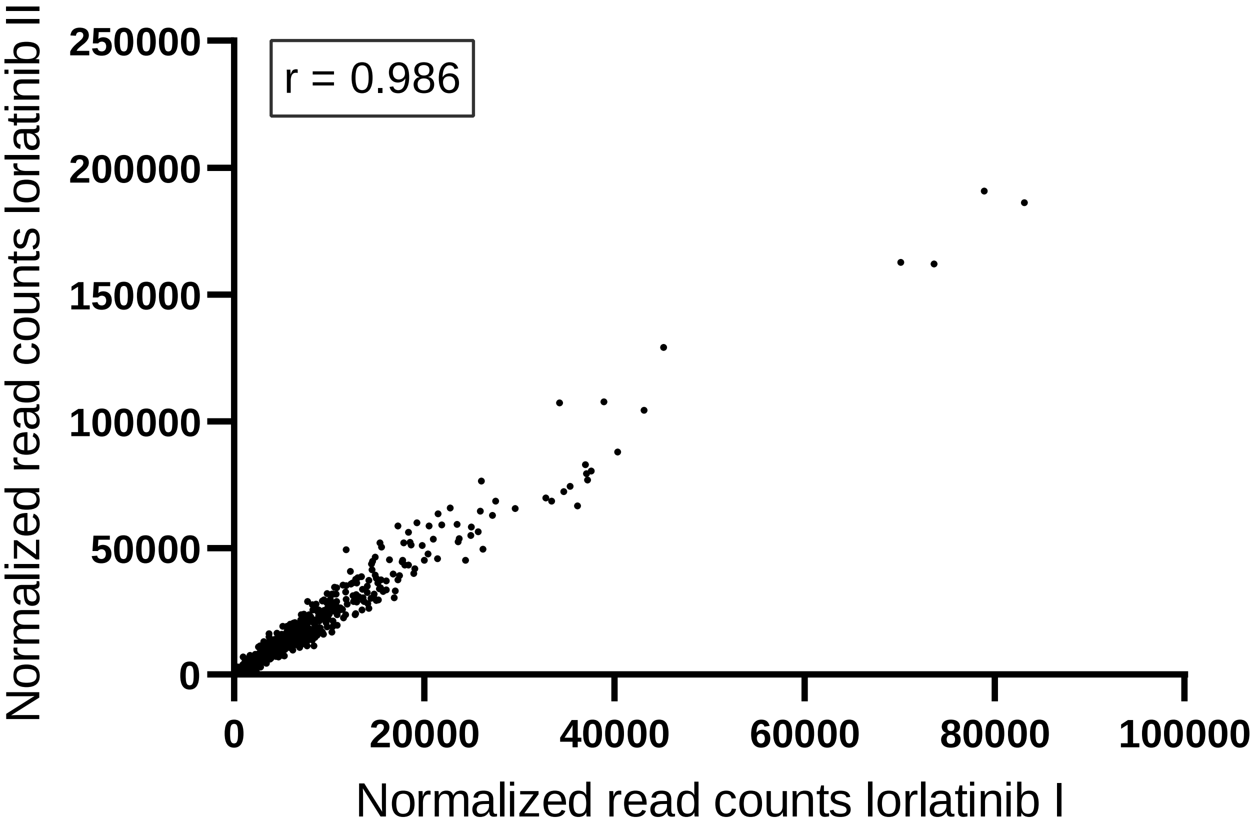
r = 0.986
Normalized read counts lorlatinib II
Normalized read counts lorlatinib I
0
50000
100000
150000
200000
250000
20000
40000
60000
80000
100000

Figure S3

a

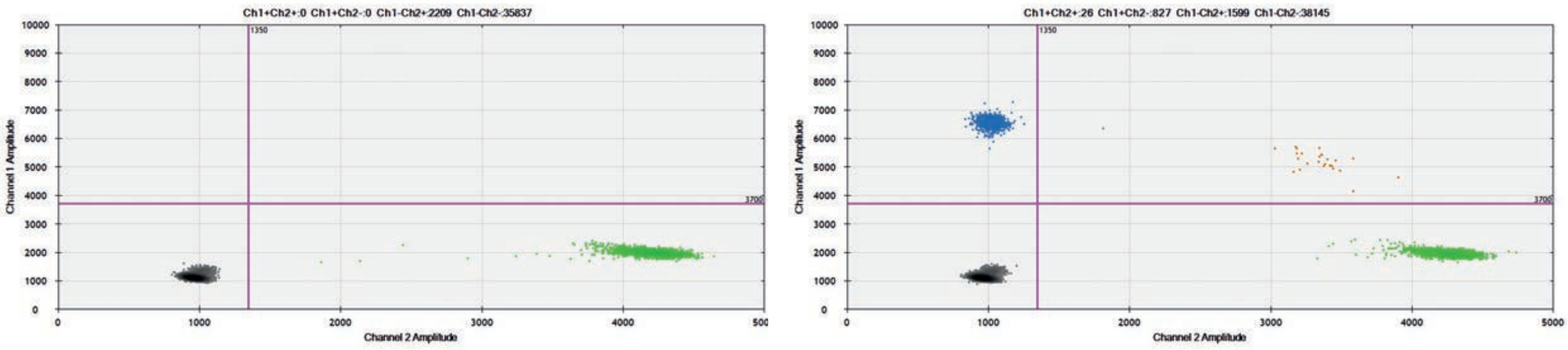

b

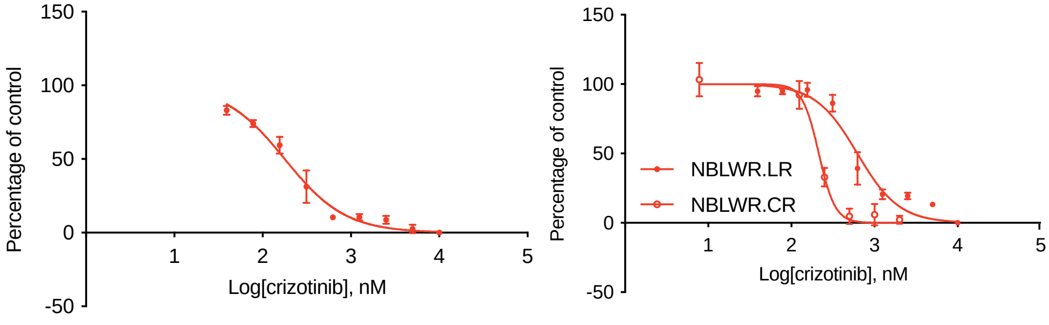

c

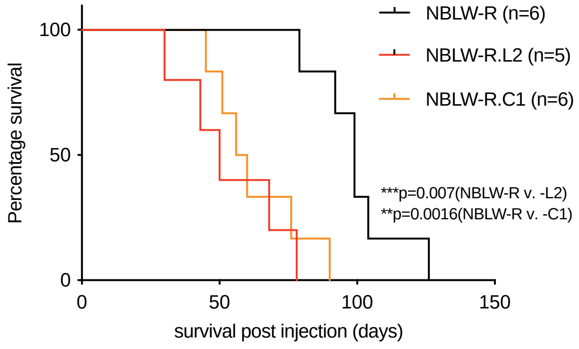

d

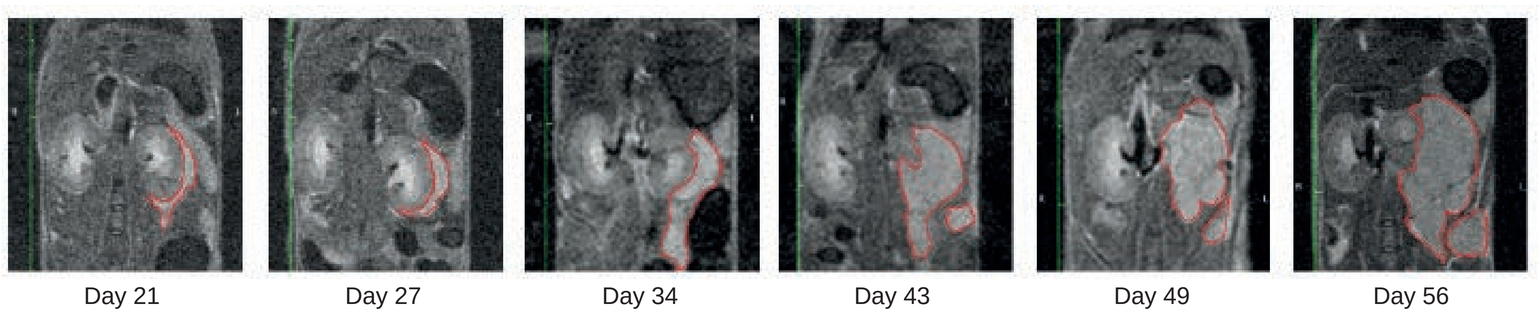

e

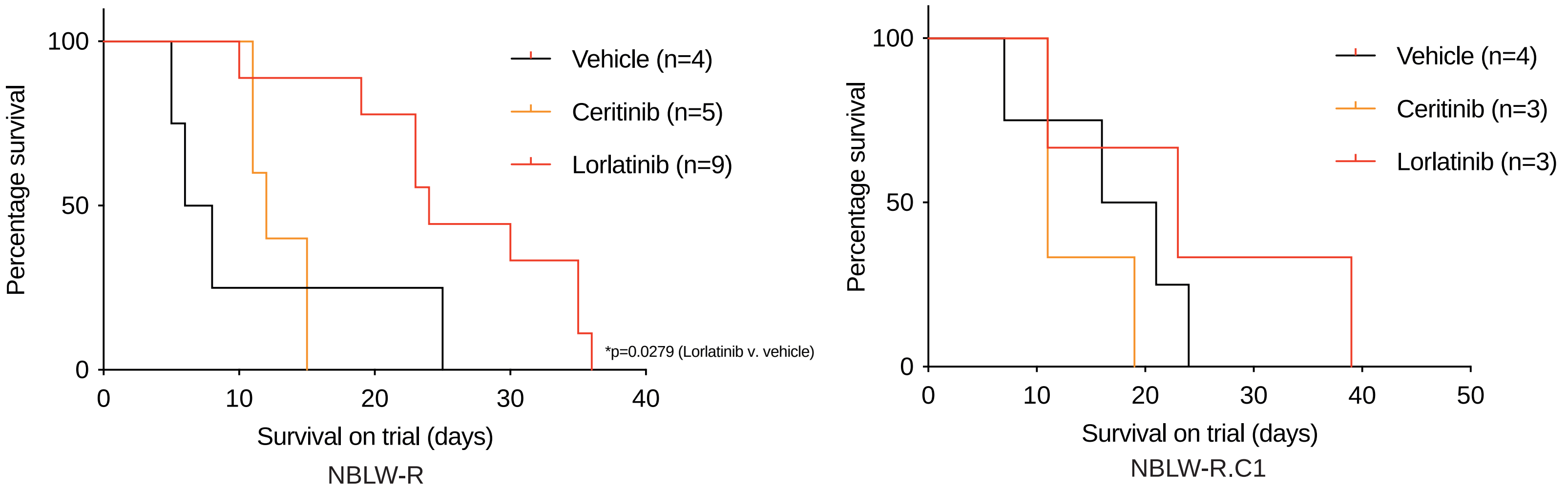

Figure S4

a

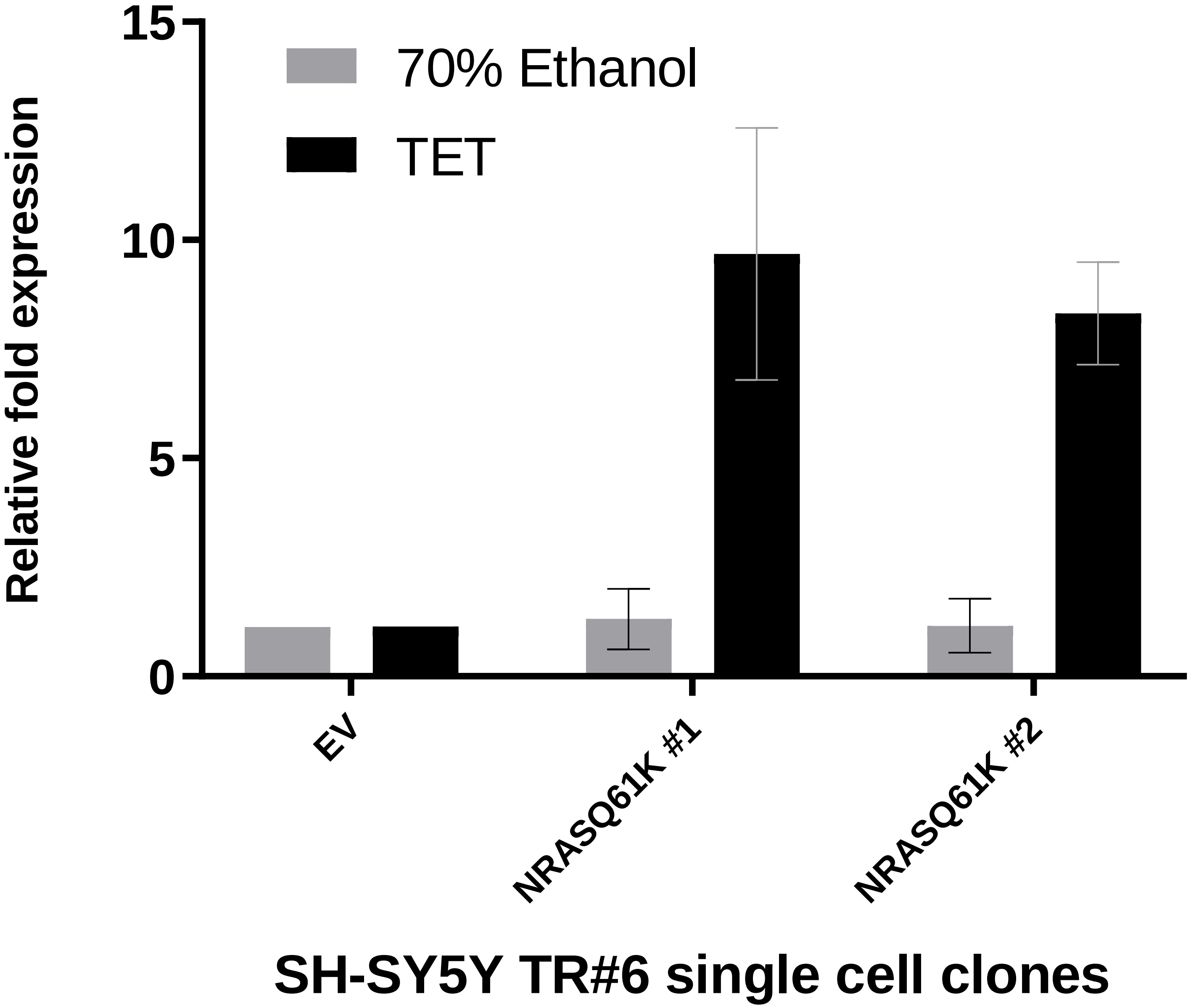

70% Ethanol
TET
Relative fold expression
15
10
5
0
EV
NRASQ61K #1
NRASQ61K #2
SH-SY5Y TR#6 single cell clones

Fig. S5

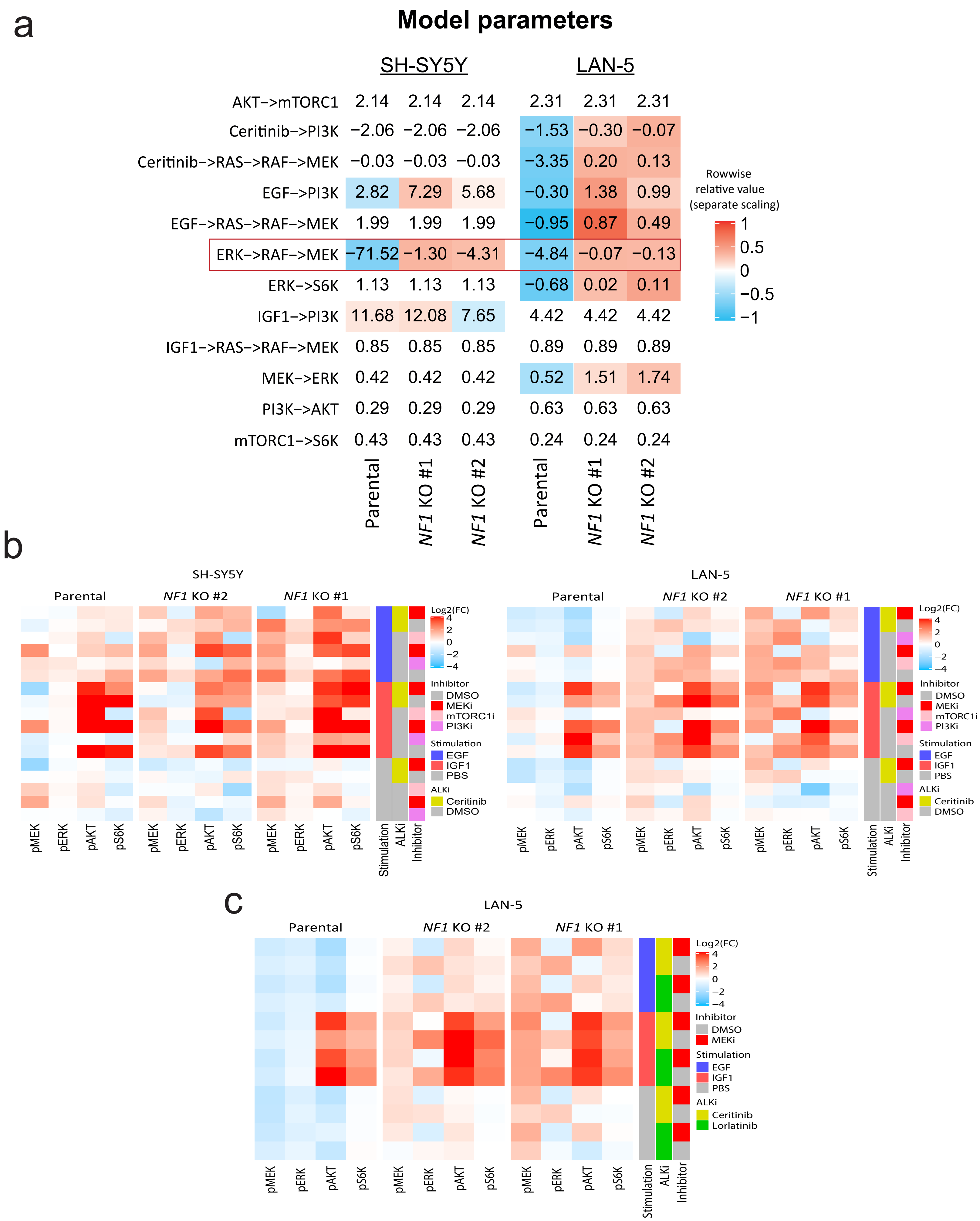
a
Model parameters
SH-SY5Y
LAN-5
AKT−>mTORC1 2.14 2.14 2.14 2.31 2.31 2.31
Ceritinib−>PI3K −2.06 −2.06 −2.06 −1.53 −0.30 −0.07
Ceritinib−>RAS−>RAF−>MEK −0.03 −0.03 −0.03 −3.35 0.20 0.13
EGF−>PI3K 2.82 7.29 5.68 −0.30 1.38 0.99
EGF−>RAS−>RAF−>MEK 1.99 1.99 1.99 −0.95 0.87 0.49
ERK−>RAF−>MEK −71.52 −1.30 −4.31 −4.84 −0.07 −0.13
ERK−>S6K 1.13 1.13 1.13 −0.68 0.02 0.11
IGF1−>PI3K 11.68 12.08 7.65 4.42 4.42 4.42
IGF1−>RAS−>RAF−>MEK 0.85 0.85 0.85 0.89 0.89 0.89
MEK−>ERK 0.42 0.42 0.42 0.52 1.51 1.74
PI3K−>AKT 0.29 0.29 0.29 0.63 0.63 0.63
mTORC1−>S6K 0.43 0.43 0.43 0.24 0.24 0.24
Parental
NF1 KO #1
NF1 KO #2
Parental
NF1 KO #1
NF1 KO #2
Rowwise relative value (separate scaling)
1
0.5
0
−0.5
−1
b
SH-SY5Y
Parental
NF1 KO #2
NF1 KO #1
LAN-5
pMEK
pERK
pAKT
pS6K
Stimulation
ALKi
Inhibitor
Log2(FC)
4
2
0
−2
−4
Inhibitor
DMSO
MEKi
mTORC1i
PI3Ki
Stimulation
EGF
IGF1
PBS
ALKi
Ceritinib
DMSO
c
LAN-5
Parental
NF1 KO #2
NF1 KO #1
pMEK
pERK
pAKT
pS6K
Stimulation
ALKi
Inhibitor
Log2(FC)
4
2
0
−2
−4
Inhibitor
DMSO
MEKi
Stimulation
EGF
IGF1
PBS
ALKi
Ceritinib
Lorlatinib

Figure S6

a

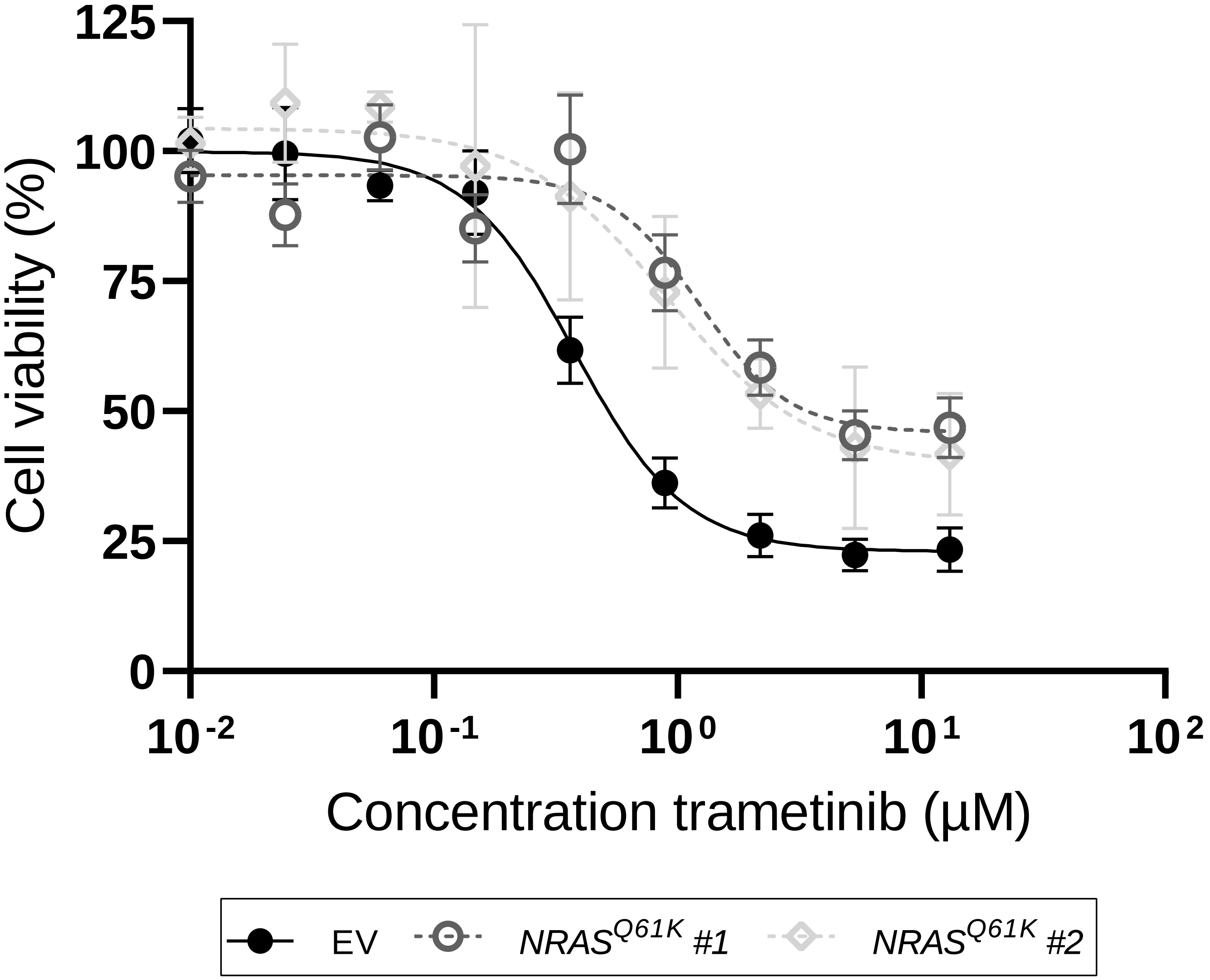

125
100
75
50
25
0
Cell viability (%)
10-2
10-1
100
101
102
Concentration trametinib (µM)
EV
NRASQ61K #1
NRASQ61K #2

Figure S7

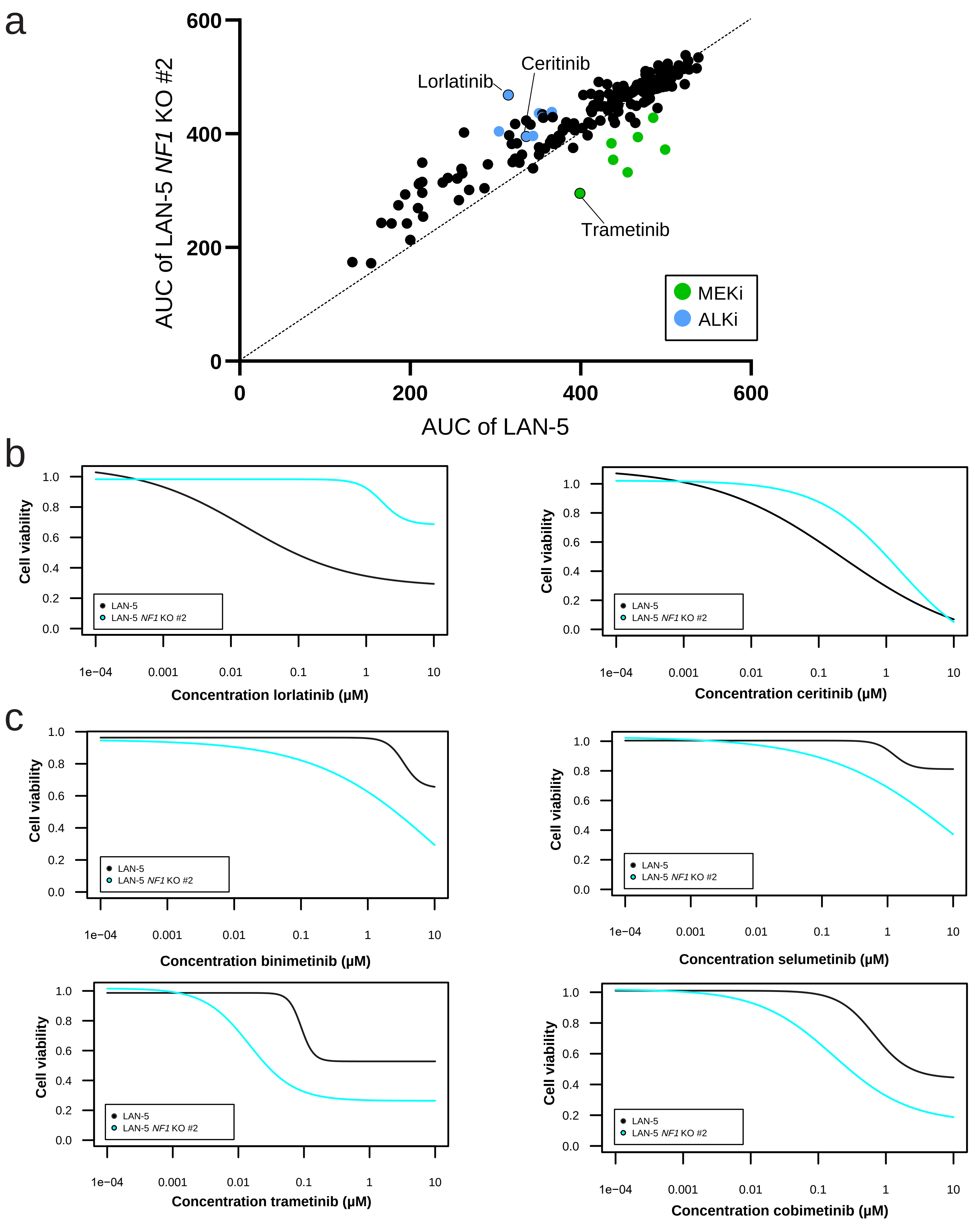
a
AUC of LAN-5 NF1 KO #2
AUC of LAN-5
0
200
400
600
Lorlatinib
Ceritinib
Trametinib
MEKi
ALKi
b
Cell viability
LAN-5
LAN-5 NF1 KO #2
Concentration lorlatinib (μM)
Concentration ceritinib (μM)
1e−04
0.001
0.01
0.1
1
10
0.0
0.2
0.4
0.6
0.8
1.0
c
Concentration binimetinib (μM)
Concentration selumetinib (μM)
Concentration trametinib (μM)
Concentration cobimetinib (μM)

SUPPLEMENTAL ITEMS TITLES AND LEGENDS

Figure S1: ALK inhibitor treatment of *ALK*-mutated neuroblastoma cell lines for optimized screening conditions (related to Figure 1).
(a) Cell viabilities of different *ALK*-mutated neuroblastoma cell lines were assessed during a 72 hours ALK inhibitor treatment with ceritinib or lorlatinib to investigate its sensitivity to ALK inhibition; values represent mean ± SD, n=3.
(b and c) Cell viability of the neuroblastoma cell line SH-SY5Y was determined during a 72 hours ALK inhibitor treatment with ceritinib or lorlatinib using an Incucyte® live-cell imaging system; values represent mean ± SD, n=3.

Figure S2: Quality control of CRISPR/Cas9 knockout screen (related to Figure 1).
(a and b) Correlation of normalized read counts per sgRNA between the two technical screen replicatesfor each ALKi respectively. Correlation coefficients (r) were calculated using Pearson correlation and tested using a two-tailed t-test; p<0.0001.

Figure S3: Lorlatinib- and Ceritinib- resistant NBLW-R neuroblastoma cells grow as aggressive tumors in the kidney capsule of nude mice (related to Figure 3).
(a) ddPCR for NRAS c.181C>A in NBLW-R bulk population (left panel) and NBLW-R.L3 bulk population (right panel). Negative represented by black droplets; wild-type NRAS represented by green droplets; NRAS c.181C>A represented by blue droplets; double positive (mutant and wild-type) represented by orange droplets. Fractional abundance of NRAS c.181C>A = 35.5 % in NBLW-R.L3 cells.
(b) 10-day GI_{50} of crizotinib in NBLW-R (178 nM) (left) and 5-day GI_{50} of crizotinib in NBLW-R.LR (621.6 nM) and NBLW-R.CR (213.9 nM) (right).
(c) Survival curve of NBLW-R (n=6) kidney capsule tumor model versus NBLW-R.L2 (n=5) and NBLW- R.C1 (n=6), following injection of 1 million cells per animal at day 1. Survival NBLW-R versus NBLW-R.L2 p=0.0007***, and NBLW-R versus NBLW-R.C1 p=0.0016** according to log-rank (Mantel-Cox) test.
(d) Series of representative MR images of an untreated NBLW-R.C1 tumor (red outline).
(e) Survival curves of NBLW-R and NBLW-R.C1 kidney capsule tumor models treated with either ceritinib (50 mg/kg), lorlatinib (10 mg/kg) or vehicle control. NBLW-R lorlatinib versus vehicle p=0.0279* according to log-rank (Mantel-Cox) test.

Figure S4: Tetracycline induced $NRAS^{Q61K}$ expression (related to Figure 4).
(a) Tetracycline induced $NRAS^{Q61K}$ expression measured using qPCR after 72 hours of tetracyline exposure (2 µg/ml). This qPCR was performed with an NRAS primer that detects wildtype NRAS and $NRAS^{Q61K}$, values represent mean ± SD, n=2, except for EV n=1.

Figure S5: Computational modeling of ALK downstream signaling using STASNet (related to Figure 6).
(a) STASNet computational modeling of ALK downstream signaling. Shown are relative values of the path with separate scaling for LAN-5 and SH-SY5Y cell lines. Negative feedback is indicated in blue. *NF1* knockout models show a weaker ERK-RAF inhibitory feedback in comparison to the respective parental cell line.
(b) Measurement and quantification of 4 phosphoproteins after perturbation with ceritinib (ALKi), trametinib (MEKi), rapamycin (mTORi), pictilisib (PI3Ki) or DMSO and subsequent stimulation with EGF, IGF or PBS (carrier) of *NF1* knockout models and respective parental lines. Values are shown as log2(fold change) to PBS+DMSO control. *NF1* knockout cell lines show increased RAS-MAPK signaling in comparison to the respective parental cell line.
(c) Measurement and quantification of 4 phosphoproteins after perturbation with ceritinib (ALKi), lorlatinib (ALKi), trametinib (MEKi), a combination of ALKi and MEKi or DMSO and subsequent stimulation with EGF, IGF or PBS (carrier) of LAN-5 *NF1* knockout models and respective parental line. Values are shown as log2(fold change) to PBS+DMSO control. Cell lines show a similar response to different ALK inhibitors.

Figure S6: MEK inhibitor treatment of ectopic NRAS^{Q61K} expression models (related to Figure 7).
(a) Cell viabilities of ectopic NRAS^{Q61K} expression models were assessed after 72 hours of MEK inhibitor exposure with trametinib using an Incucyte® live-cell imaging system to investigate sensitivities to MEK inhibition; values represent mean ± SD, n=3

Figure S7: High-throughput drug screening of LAN-5 and LAN-5 *NF1* KO#2 clone (relatedto Figure 7)
(a) Comparison of area under the curve (AUC) values derived from a high-throughput drug screen of the parental LAN-5 cell line and the LAN-5 *NF1* KO #2 clone using a compound library composed of 197 drugs. ALK inhibitors are highlighted in blue and MEK inhibitors are colored in green. A higher AUCvalue describes a less sensitive phenotype.
(b) Cell viabilities of parental LAN-5 and the LAN-5 *NF1* KO #2 clone were assessed after a 72 hour drug exposure to ALK inhibitors using the MTT assay as part of a high-throughput drug screen to investigate new collateral sensitivities of *NF1* KO cell lines.
(c) Cell viabilities of parental LAN-5 and the LAN-5 *NF1* KO #2 clone were assessed after a 72 hour drug exposure to MEK inhibitors using the MTT assay as part of a high-throughput drug screen to investigate new collateral sensitivities of *NF1* KO cell line

Table S1.| Full clinical data on neuroblastoma patients

	Patient #1	Patient #2	Patient #3	Patient #4
**Diagnosis**	stage 4 high risk NB	stage 4 high risk NB	stage 4 high risk NB	stage 4 high risk NB
**Primary therapy**	according to NB 2004 HR	according to NB 2004 HR	according to SIOPEN HR NBL	according to NB 2004 HR
**First relapse**	*15 months after diagnosis*<br>• *disseminated disease*	*63 months after diagnosis*<br>• bone marrow metastasis<br>• Bone metastasis right Ulna	*9 months after diagnosis*<br>• metastatic skull site	*18 months after diagnosis*
**Timepoint of first relapse biopsy**	*15 months after diagnosis*<br>• Biopsy of first relapse in right adrenal gland<br>• panel sequencing<br>– ALK p.F1174L (c.3522C>A)	*63 months after diagnosis*<br>• panel sequencing<br>– ALK p.R1275Q (c.3823C>T)<br>– MYCN p.P44L	*9 months after diagnosis*<br>• Biopsy: ALK p.R1275Q (c.3823C>T)<br>*15 months after diagnosis*<br>*Biopsy before Lorlatinib therapy*<br>• Liquid biopsy on ctDNA<br>– ALK p.R1275Q (c.3823C>T)	*58 months after diagnosis*<br>Tumor biopsy<br>Analysed using whole-exome sequencing:<br>• ALK p.R1275Q (c.3823C>T)
**Salvage therapy before ALK targeted therapy**	• Anti-GD2 antibody therapy and chemotherapy	• Therapy according to RIST protocol	Cyclophosphamide and topotecan<br>*stable disease*	• Therapy according to RIST protocol<br>• radiation 39.6Gy, Carboplatin, ^{131}I-mIBG therapy, High-dose chemotherapy regimes BuMel, autologous stem cell transplantation, anti-GD2 antibody (Dinutuximab) + interleukin 2 (IL2)+ 13-cis retinoic acid<br>• Metronomic therapy<br>• Metronomic therapy, radiation

**ALK targeted therapy**	Ceritinib	Ceritinib	*12 months after diagnosis*<br><br>Ceritinib<br>*initial visible shrinkage*<br><br>*15 months after diagnosis*<br><br>Lorlatinib<br>*evidence of tumour growth*	*41 months after diagnosis*<br><br>*Ceritinib*<br><br>*44 months after diagnosis*<br><br>• *Ribociclib, Ceritinib*<br>• *Ceritinib,*<br>*Haplo stem cell transplantation, anti-GD2 antibody (Dinutuximab) + IT*<br><br>*58 months after diagnosis*<br><br>• *Lorlatinib, Ribociclib, Temodal*<br>• *Lorlatinib, Temodal, MIBG*
**Timepoint of biopsy after ALK targeted therapy**	*22 months after diagnosis*<br><br>• panel sequencing<br>– ALK p.F1174L (c.3522C>A)<br>– NF1 p.A320fs (c.960_961delTG)<br>– NF1 p.F1593S (c.4778T>C)	*77 months after diagnosis*<br><br>• panel sequencing<br>- ALK p.R1275Q (c.3823C>T)<br>- NF1 p.R1276*<br>-MYCN p.P44L	*20 months after diagnosis*<br><br>• Liquid biopsy on ctDNA<br>– NRAS p.Q61K<br>– ALK p.R1275Q (c.3823C>T)	*63 months after diagnosis*<br><br>Tumor biopsy:<br>• Analysed using whole-exome sequencing<br>– ALK p.R1275Q (c.3823C>T)<br>– HRAS p.Q61K
**Best response to ALK targeted therapy**	partial response	partial response	partial response	partial response
**Further therapy**	Therapy according to RIST protocol	Anti-GD2 antibody therapy and chemotehrapy	Trametinib and Debrafinib therapy	
**Further course of disease**	*27 months after diagnosis*<br><br>death	*80 months after diagnosis*<br><br>death	*22 months after diagnosis*<br><br>death	*64 months after diagnosis*<br><br>death
