# Supplementary material for: Mutations in ALK signaling pathways conferring resistance to ALK inhibitor treatment lead to collateral vulnerabilities in neuroblastoma cells
Source: Mol Cancer. 2022 Jun 10;21:126. doi: 10.1186/s12943-022-01583-z (PMC9185889; doi:10.1186/s12943-022-01583-z)
Supplement: Supplementary file 6 — Additional file 6. Supplementary Materials and Methods. [file 12943_2022_1583_MOESM6_ESM.pdf]

Additional file 6 Supplementary Materials and Methods

Plasmid library amplification

For the CRISPR/Cas9-based negative selection screens the human CRISPR knockout library Brunello was used as a one-vector system (Addgene, #73179) (1). The pooled plasmid library targeting 19 114 genes with 76 441 gRNAs (average of 4 gRNAs per gene) was amplified as described elsewhere (1). In brief, the lentiCRISPRv2 plasmid library was amplified by electroporation into STBL4 ™ (Invitrogen, #11635-018) using 100 ng plasmid DNA (pDNA) and 50 µl STBL4 cells. 25 µl of this mixture were pipette into the recommended cuvettes and electroporation performed with the recommended settings by the manufacturer. Cell suspension was incubated for 1 h at 30 °C and subsequently 6x 100 ml LB media (Carl Roth, #X968.2) supplemented with 50 µg/ml ampicillin (Carl Roth, #K029.2) inoculated in 1 l flasks and incubated at 30 °C overnight. Plasmid DNA was isolated using the NucleoBond PC 10000EF kit (Macherey-Nagel, #740548) according to manufacturer instructions. The amplified plasmid pool was PCR amplified and send for sequencing to verify representation of sgRNA in the plasmid pool as described in the main methods section.

Lentivirus production and transduction

For production of library containing lentivirus transfection into $20*10^6$ HEK293FT cells (Invitrogen, #R70007) was performed using the CalPhos™ Mammalian Transfections Kit (Takara, #631312) according to protocol. The following plasmids were used in a 1:2:2:3 ratio pMD2.G (Addgene, #12259), pMDLg/pRRE (Addgene, #12251), pRSV-REV (Addgene, #12253), and lentiCRISPRv2 plasmid library. After approximately 16 hours the medium was changed and supplemented with a final sodium butyrate (Sigma, #B5887) concentration of 6 mM. Virus was harvested 48 hours after sodium butyrate treatment. Supernatant was filtered using stericup durapore 0.45 µm (Millipore, #SCHVU05RE) and ultracentrifugated at 24 500 rpm,4 °C for 1 hour and 30 minutes. Virus titer determination was performed by transducing SH-SY5Y cells with different volumes of virus and 5 µg/ml polybrene (Millipore, #TR-1003-G). Medium was changed to puromycin selection medium (0.8 µg/ml puromycin,

Thermo Fisher, #A1113803) 48 hours after transduction to select for infected cells. Cells were kept in selection medium until all cells in the control well were dead. Cells were stained using crystal violet (Sigma, #C0775-25) and colonies counted to determine the virus titer.

Generating and validating knockout cell lines

In order to introduce *NF1* knockouts in respective cell line single gRNAs were designed to target exon1 (CTCGTCGAAGCGGCTGACCA and CGCGCACAGGCCGGTGGAAT) and exon 30 (TCATCTGAAGGAGGTTCCGC and TGGCACACACTTCGAAGTTG) of *NF1* (additional file 5 Table S4) using the web tool CHOPCHOP(2). Oligos were ordered with 5’-CACC-3’ or 5’-AAAC-3’ overhangs from Eurofins. Knockout cell lines were generated as described elsewhere (3). In brief, backbone pSpCas9(BB)-2A-Puro (PX459) (Addgene, #48139) was digested with *Bbs*I (NEB, #R0539S) and gel extracted (Qiagen, QIAquick gel extraction kit, #28704). SgRNA oligos were phosphorylated and annealed using T4 polynucleotide kinase (NEB, #M0201) and incubation at 37 °C for 30 minutes, 5 minutes at 95 °C and ramp down to 25 °C at 5 °C min^{-1}. Backbone and each sgRNA were ligated using T4 DNA ligase (NEB, #M0202S). After construct transformation and plasmid DNA isolation, plasmids were validated using restriction digest and sanger sequencing. Different neuroblastoma cell lines were plated and transfected with each plasmid using Lipofectamine™ LTX (ThermoFisher, #15338030) according to manufacturer instructions. After 24 hours the medium was changed and supplemented with 0.7 µg/ml puromycin (Thermo Fisher, #A1113803) to select for positively selected cell clones until all cells in control wells were dead. Clonal cell lines were isolated by dilution and expanded. Single cell clones were validated using western blot, T7 assay and PCR (KAPA2G Fast HotStart ReadyMix, KAPA BIOSYSTEMS, #KK5601, 95 °C for 5 min; 95 °C for 10 s,62 °C for 15 s,72 °C for 10 s,72 °C 5 min for 35 cycles) with subsequent sanger sequencing. The sequences of the primers used for PCR analyses are described in additional file 5 Table S4.

Generating NRAS^{Q61K} expression models

A validated SH-SY5Y TR cell clone (transfected once with pcDNA6/TR from Invitrogen, #V102520) was transfected using Lipofectamine 3000 (Invitrogen, #L3000001) with the vector pT-Rex-DEST30-NRAS^{Q61K} according to manufacturer's instructions. That vector was generated by Gateway cloning using LR clonase with entry clone pDONR223_NRAS_q.61k (Addgene, #81657) and destination vector pT-Rex-DEST30 (Invitrogen, #12301016) according to manufacturer's instructions. 48 hours after transfection positive cells were selected using 0.4 mg/ml G418 (Genaxxon, #M3118.0100). Selected SH-SY5Y TR NRAS^{Q61K} cells were used for serial dilution to isolate clonal cell lines. RT-qPCR and western blotting were used to validate single cell clones as described below. For tetracycline induced expression of NRAS^{Q61K} cells were exposed to 2 µg/ml tetracycline (Sigma Aldrich, #T7660) for 48 hours (or for 72 hours for initial clone validation).

Panel sequencing of resistant NBLW-R cells

Library preparation was performed using the KAPA HyperPlus Kit (Roche, #07962380001) and SeqCap EZ adapters (NimbleGen), following the manufacturer's protocol, including dual-SPRI size selection of the libraries (250-450 bp). 1µg of the pooled library DNA was hybridized to a custom panel of 92 genes (473kb) (NimbleGen SeqCap EZ library, Roche). Sequencing was performed on an Illumina NextSeq500 to produce 75bp, paired-end reads as dictated by the number of samples in the pools and a target coverage of 400x. The analysis has been performed using Molecular Diagnostics Information Management System v4.0 (Developed by the Clinical Genomics at the Royal Marsden) based on genome build hg19. Raw data was converted to FASTQ files using the bcl2fastq2 software, FASTQs were then aligned to the hg19 reference genome using BWA-MEM (4)and quality metrics are generated using Picard (https://broadinstitute.github.io/picard/). Single nucleotide variants are called using Mutect2 (5) and are annotated using PCGR (6). For tumor samples structural variants are calling using the Manta software (7) and are annotated AnnotSV (8, 9). CNVs were called using a proprietary tool developed by the Clinical Genomics team at the Royal Marsden.

Protein lysate preparation and Western Blot

For protein lysate preparation cell pellets were resuspended in lysis buffer (15mM HEPES, 150mM NaCl, 10mM EDTA, 2% Triton X-100, pH7.5) (10) supplemented with PhosSTOP (Roche, #4906845001) and cOmplete Mini EDTA free protease inhibitor (Roche, #11836170001). Samples were incubated for 30 min on ice, vortexed for 10s and centrifuged for 30min at 4°C. Protein concentrations were determined using the Pierce™ BCA Protein assay kit (ThermoFisher, #23225) according to manufacturer instructions. For detection of NF1 a wet blot was performed. Therefore, 10-15µg of samples were supplemented with 4x Laemmli buffer (Biorad, #161-0747) containing 355mM β-mercaptoethanol and incubated at 95°C for 5 minutes. Samples were loaded on NuPAGE 3-8% Tris-Acetate Protein Gels (Life technologies, #EA0375) and electrophoresis performed in NuPAGE Tris-Acetate SDS Running Buffer (20x) (Life technologies, #NP0007) supplemented with NuPAGE Antioxidant (Life technologies, #NP0005) at 80 V for 3hours. HiMark Pre-stained Protein Standard (Life technologies, #LC5699) as well as PageRuler™ Prestained Protein Ladder (Thermo Scientific, #26617) were used. Wet blots were performed using 0.2µm PVDF membranes (Roche, #3010040001) and a wet blot transfer buffer (25 mM Tris, 192 mM glycine, pH 8.3, 10% Methanol, 0.05% SDS) at 4°C, 30V for 16hours. Smaller proteins were separated using 10% Tris-Glycine-SDS polyacrylamide gels and a 1x TRIS-Glycine SDS running buffer (25mM Trizma base, 192mM Glycine,1%SDS). Subsequent semi-dry blots were performed using 1x transfer buffer (25mM Trizma base, 192mM Glycine,20% methanol) at 25V for 30min. Membranes were blocked in 10% milk in TBS-T or for phosphoprotein detection in 10%BSA in TBS-T. Membranes were incubated with respective antibodies diluted in 5% milk-TBS-T or 5% BSA TBS-T over night at 4°C. Incubation with secondary antibodies was performed for 1hour and 30 minutes at room temperature. Antibodies used for detection during experiments in this manuscript were NF1 1:500 (CST, #14623), NF1 1:3000 (Abcam, #17963), MEK ½ 1:1000 (CST, #9122), pMEK ½ 1:1000 (CST, #9121), ERK1/2 1:1000 (CST, #4695), pERK1/2 1:1000 (CST, #4370), AKT 1:1 000 (CST, #2938), pAKT 1:1000 (CST, #4058), β-Actin 1:1000 (Santa Cruz, #sc-47778), Vincullin 1: 1 000 (CST, #4650) CST, anti-mouse 1:5 000 (Dianova , #115-035-003), anti-rabbit 1: 5 000 (Dianova , #111-035-

003) and NRAS(F155) 1:100 (Santa Cruz, #sc-31). Blots were developed using a western blotting luminol reagent (Santa Cruz, #sc-2048) and documented with a Fusion FX imaging system (VILBER).

Immunoassays for the quantification of ALK and phosphorylated ALK

The immunoassays for quantification of ALK and phosphorylated ALK was described elsewhere (11). Briefly, neuroblastoma cells were lysed in 1% CHAPS, and protein quantification undertaken using Direct Detect™ (Millipore). Lysates were plated in duplicate into a multi-array 96-well plate (MSD), pre-coated with anti-ALK antibody 31F12 (Cell Signaling Technology), and pre-blocked in 5% bovine serum albumin. Following overnight incubation at 4°C the plate was washed (TBST) and incubated for one hour in either anti-ALK D5F3 or anti-pY1586ALK 3B4 (Cell Signaling Technology. After a further wash (TBST), the plate was incubated with Sulfo-Tag™ Anti-Rabbit Antibody (MSD) for another hour, before a final wash (TBST), and addition of 2x Read Buffer (MSD) to each well. The plate was read on an Meso QuickPlex SQ 120 (MSD).

In vivo studies

All *in vivo* experimental protocols were monitored and approved by the ICR Animal Welfare and Ethical Review Body, in compliance with guidelines specified by the UK Home Office Animals (Scientific procedures) Act 1986 and the United Kingdom National Cancer Research Institute Guidelines for the Welfare of Animals in Cancer Research (12).Female CrTac:NU (NCR)-*Foxn1*^{nu} mice at 6 weeks of age (weight >20g) were purchased from Charles River for the orthotopic implantation of NBLW-R and NBLW-R resistant lines. Two to five mice were housed in accordance with Home Office Code of Practice in individually ventilated cages, provided with standard irradiated laboratory diet and sterile water *ad libitum*, in a controlled temperature environment. Specific-pathogen free (SPF) conditions were maintained with health monitoring conducted quarterly to a Federation of European Laboratory Animal Science Associations (FELASA) standard profile. For the survival study (Figure S3C and D) 1 million cells were injected into the kidney capsule in 5μl of matrigel under aseptic conditions. The survival of the animal was recorded

following injection, according to UK Home Office license limits. Changes in tumor volume were monitored using MRI on a 7T horizontal bore MicroImaging system (Bruker Instruments) using a 3cm birdcage coil. Anatomical T_2-weighted coronal images were acquired through the mouse abdomen. For the treatment survival studies (Figure S3E), 1 million cells were injected into the kidney, as above. Tumor development was monitored by palpation, and at palpation of a 5mm tumor, animals were randomly assigned to receive dosing with either vehicle (0.5 % methylcellulose, 0.5 % Tween80 in water), ceritinib 50 mg/kg orally, daily, or lorlatinib 10 mg/kg orally, daily. The survival was recorded from the day of first dosing until the animal was culled according to UK Home Office license limits. Samples were collected 2 hours following the final dose.

Whole-exome sequencing

Before using cell lines like LAN-5 for generating *NF1* knockout models, cells were sent for whole-exome sequencing to exclude the presence of mutations in ALK downstream signaling pathways. Library preparation of the genomic DNA was performed according to the Agilent Low Input Exome-Seq Human v7 protocol at the DKFZ Genomic and Proteomics core facility, High-throughput sequencing unit in Heidelberg. Library was sequenced on an Illumina HiSeq 4000 as a paired-end 100bp run. Sequencing data has been aligned to the hg19 version of the reference genome using the Burrows–Wheeler Aligner MEM v1.10. Read duplicates were marked with biobambam2 v2.0.87. Small indels and Structural Variants were detected using SvABA v1.1.0 (13) and Mutect2 (GATK)v4.1.9.0 (5) was used to detect single-nucleotide variants. Copy number variants were detected using Control-FREEC v11.6 (14).

High-throughput drug screen

Screening experiments and processing were performed by the high-throughput screening facility of the Princess Máxima Center (https://research.prinsesmaximacentrum.nl/en/core-facilities/high-throughput-screening). The drug library used contains 197 drugs, whereof 193 are dissolved in DMSO and stored at room temperature under nitrogen atmosphere; four drugs (metformin, perifosine,

carboplatin and oxaliplatin) are dissolved in MilliQ-water and one (cisplatin) is dissolved in a saline solution, which are stored at -20 °C. Before the high-throughput screen, the 384-well working plates (384LDV-Plates, Labcyte, # LP-0200) containing the dissolved drugs were shaken (30 min at rt) and centrifuged (1 min at 1500 rpm). Subsequently, the working plates were surveyed with the Echo 550 dispenser (Beckman Coulter) to determine whether the amount of solution in the wells is sufficient to start the screen (minimal 2.5 µl) and the DMSO percentage is >80%. For the screen performance cells (5000 cells/well) were seeded in 384-well microplates (Corning, #3764) using the Multidrop™ Combi Reagent Dispenser (Thermo Scientific). After overnight incubation, the drugs were added in duplicate using the Echo 550 dispenser (Beckman Coulter). The final concentration of solvent was 0.25%. The final drug concentrations ranged standard between 0.1 nM and 10 µM, although some drugs were added on lower and higher concentrations additionally. Cells treated with only DMSO were used as positive controls, whereas cells treated with staurosporine (final concentration of 10 µM) were used as negative controls. After 72 hours of treatment at standard culturing conditions, cell viability was measured using the 3-(4,5-dimethylthiazol-2yl)-2,5-diphenyltetrazolium (MTT) assay (15).

The half maximal concentration that inhibits the viability (IC_{50}) and the area-under-the-curve (AUC) of the standard concentration range was calculated by determining the concentrations of the drug needed to achieve a 50% reduction in cell viability, after normalizing the data to the DMSO-treated cells (defined as 100% viability) and the empty controls (0% viability) using the extension package *drc* in the statistic environment of R Studio (version 4.0.2) (16). Quality of the screens was approved after assessment of the cell growth (absorbance signal of t_{72}/t_0), the negative, positive, and empty controls and the amount of variability between the duplicates.

Droplet digital PCR of NBLW-R cells

Droplet digital PCR (ddPCR) was performed using the QX200 Droplet Digital PCR System (Bio-Rad). A custom TaqMan SNP genotyping assay for NRAS c.181C>A was purchased from Thermo Fisher (Thermo Fisher, #4331349). 5 ng of DNA or NGS library preparation was added to a total volume of 22 µl in a

ddPCR reaction containing 11 µl ddPCR Supermix for Probes (Bio-Rad, #1863024) and 0.55 µl of 40x concentrated ddPCR assay. The final concentration of the primers and probes in the reactions was 900 nM and 200 nM respectively. The ddPCR mixes were partitioned into ~20,000 droplets per well on an Automated Droplet Generator (Bio-Rad) prior to PCR on a Veriti thermal cycler (Thermo Fisher) using the protocol: 95 °C for 10 min, followed by 40 cycles of 94 °C for 30 s and 57 °C for 1 min, prior to a final extension at 98 °C for 10 min. The temperature ramp rate was set to 2 °C/s for all steps. Each sample or library preparation was run in duplicate. A positive control, negative control and a no template control were also run using the same conditions. The amplified ddPCR reactions were kept at 4 °C before being read on a QX200 Droplet Reader (Bio-Rad). The ddPCR data was analyzed using QuantaSoft v1.7.4 software. The variant was accepted as being detected if there was ≥2 variant-positive droplets.

Panel sequencing of tumor samples

Tumor material from patients treated in Berlin was analyzed using a hybrid-capture based next generation panel sequencing approach as described elsewhere (17).

Panel sequencing of cfDNA samples

One mL of plasma per sample was available and used for cfDNA (circulating free DNA) extraction. Full AVENIO ctDNA (circulating tumor DNA) workflow from extraction to sequencing was used, following manufacturer's protocol at the Clinical Genomics lab in the Royal Marsden Hospital. This kit uses commercial hybrid-capture panel (AVENIO ctDNA Expanded kit, Roche, #08061076001). Sequencing was performed on an Illumina NextSeq500 instrument and High Output 150bp paired-end sequencing (Illumina, #20024907), for a total of 8 samples. Samples output over 45 million reads each, median unique depth of >5000 fold. Analysis was performed using Roche AVENIO App server, Analysis Software Version: 1.1.0.

References

1. Doench JG, Fusi N, Sullender M, Hegde M, Vaimberg EW, Donovan KF, et al. Optimized sgRNA design to maximize activity and minimize off-target effects of CRISPR-Cas9. Nat Biotechnol. 2016;34(2):184-91.
2. Labun K, Montague TG, Gagnon JA, Thyme SB, Valen E. CHOPCHOP v2: a web tool for the next generation of CRISPR genome engineering. Nucleic Acids Res. 2016;44(W1):W272-W6.
3. Ran FA, Hsu PD, Wright J, Agarwala V, Scott DA, Zhang F. Genome engineering using the CRISPR-Cas9 system. Nat Protoc. 2013;8(11):2281-308.
4. Li H. Aligning sequence reads, clone sequences and assembly contigs with BWA-MEM. arXiv. 2013;preprint arXiv:1303.3997v2
5. Benjamin D, Sato T, Cibulskis K, Getz G, Stewart C, Lichtenstein L. Calling Somatic SNVs and Indels with Mutect2. bioRxiv. 2019:861054.
6. Nakken S, Fournous G, Vodák D, Aasheim LB, Myklebost O, Hovig E. Personal Cancer Genome Reporter: variant interpretation report for precision oncology. Bioinformatics. 2017;34(10):1778-80.
7. Chen X, Schulz-Trieglaff O, Shaw R, Barnes B, Schlesinger F, Källberg M, et al. Manta: rapid detection of structural variants and indels for germline and cancer sequencing applications. Bioinformatics. 2015;32(8):1220-2.
8. Geoffroy V, Guignard T, Kress A, Gaillard J-B, Solli-Nowlan T, Schalk A, et al. AnnotSV and knotAnnotSV: a web server for human structural variations annotations, ranking and analysis. Nucleic Acids Res. 2021;49(W1):W21-W8.
9. Geoffroy V, Herenger Y, Kress A, Stoetzel C, Piton A, Dollfus H, et al. AnnotSV: an integrated tool for structural variations annotation. Bioinformatics. 2018;34(20):3572-4.
10. Hecht M, Schulte JH, Eggert A, Wilting J, Schweigerer L. The neurotrophin receptor TrkB cooperates with c-Met in enhancing neuroblastoma invasiveness. Carcinogenesis. 2005;26(12):2105-15.
11. Tucker ER, Tall JR, Danielson LS, Gowan S, Jamin Y, Robinson SP, et al. Immunoassays for the quantification of ALK and phosphorylated ALK support the evaluation of on-target ALK inhibitors in neuroblastoma. Mol Oncol. 2017;11(8):996-1006.
12. Workman P, Aboagye EO, Balkwill F, Balmain A, Bruder G, Chaplin DJ, et al. Guidelines for the welfare and use of animals in cancer research. British Journal of Cancer. 2010;102(11):1555-77.
13. Wala JA, Bandopadhayay P, Greenwald NF, O'Rourke R, Sharpe T, Stewart C, et al. SvABA: genome-wide detection of structural variants and indels by local assembly. Genome Res. 2018;28(4):581-91.
14. Boeva V, Popova T, Bleakley K, Chiche P, Cappo J, Schleiermacher G, et al. Control-FREEC: a tool for assessing copy number and allelic content using next-generation sequencing data. Bioinformatics. 2012;28(3):423-5.
15. Twentyman PR, Luscombe M. A study of some variables in a tetrazolium dye (MTT) based assay for cell growth and chemosensitivity. British journal of cancer. 1987;56(3):279-85.
16. Ritz C, Baty F, Streibig JC, Gerhard D. Dose-Response Analysis Using R. PLOS ONE. 2016;10(12):e0146021.
17. Szymansky A, Kruetzfeldt L-M, Heukamp LC, Hertwig F, Theissen J, Deubzer HE, et al. Neuroblastoma Risk Assessment and Treatment Stratification with Hybrid Capture-Based Panel Sequencing. Journal of Personalized Medicine. 2021;11(8).
